# Genetic Modifiers of Chromatin Acetylation Antagonize the Reprogramming of Epi-Polymorphisms

**DOI:** 10.1371/journal.pgen.1002958

**Published:** 2012-09-20

**Authors:** Anne-Laure Abraham, Muniyandi Nagarajan, Jean-Baptiste Veyrieras, Hélène Bottin, Lars M. Steinmetz, Gaël Yvert

**Affiliations:** 1 Laboratoire de Biologie Moléculaire de la Cellule, Ecole Normale Supérieure de Lyon, CNRS, Université de Lyon, Lyon, France; 2 BioMiningLab, Lyon, France; 3 Genome Biology Unit, European Molecular Biology Laboratory, Heidelberg, Germany; Queensland Institute of Medical Research, Australia

## Abstract

Natural populations are known to differ not only in DNA but also in their chromatin-associated epigenetic marks. When such inter-individual epigenomic differences (or “epi-polymorphisms”) are observed, their stability is usually not known: they may or may not be reprogrammed over time or upon environmental changes. In addition, their origin may be purely epigenetic, or they may result from regulatory variation encoded in the DNA. Studying epi-polymorphisms requires, therefore, an assessment of their nature and stability. Here we estimate the stability of yeast epi-polymorphisms of chromatin acetylation, and we provide a genome-by-epigenome map of their genetic control. A transient epi-drug treatment was able to reprogram acetylation variation at more than one thousand nucleosomes, whereas a similar amount of variation persisted, distinguishing “labile” from “persistent” epi-polymorphisms. Hundreds of genetic loci underlied acetylation variation at 2,418 nucleosomes either locally (in *cis*) or distantly (in *trans*), and this genetic control overlapped only partially with the genetic control of gene expression. *Trans*-acting regulators were not necessarily associated with genes coding for chromatin modifying enzymes. Strikingly, “labile” and “persistent” epi-polymorphisms were associated with poor and strong genetic control, respectively, showing that genetic modifiers contribute to persistence. These results estimate the amount of natural epigenomic variation that can be lost after transient environmental exposures, and they reveal the complex genetic architecture of the DNA–encoded determinism of chromatin epi-polymorphisms. Our observations provide a basis for the development of population epigenetics.

## Author Summary

Chemical modifications of chromatin, such as DNA methylation, incorporation of histone variants, or post-translational modifications of histone proteins, constitute the “epigenome” and confer specific properties to genome functions. Epigenomes differ from one individual to another, opening the exciting perspective to decipher the origins of these differences and their impact on physiology. However, population epigenomics remains challenging because, unlike DNA mutations, epigenetic hallmarks are themselves regulated. They can change upon particular environmental conditions, they may be inherited epigenetically, or they may result from activities encoded in the DNA. Thus, estimating the stability of intra-species epigenomic variation and its dependence on DNA polymorphisms is essential. Using a chemical perturbation of yeast cells as an experimental model system, we found that acetylation variation was persistent at some nucleosomes and labile at other nucleosomes. By studying a segregating population, we mapped DNA polymorphisms that affected chromatin acetylation levels at numerous nucleosomes. Strikingly, nucleosomes showing persistent variation of acetylation corresponded to those for which acetylation was under genetic control. Thus, part of epigenomic variation is stabilized by a DNA–encoded determinism, and another part can be reprogrammed if environmental perturbations are experienced. These results provide a necessary basis for upcoming developments in population epigenomics.

## Introduction

Recent studies have shown that individuals largely differ in their epigenomic chromatin signatures. This finding makes tracking epigenetic marks in natural populations attractive, including investigating their possible contribution to the variation of common physiological traits. So far, epigenomic intra-species diversity has been primarily studied at the level of the methylome (DNA methylation profile). Natural accessions of *A. thaliana* were found to differ in their methylation level at about 10% of all CCGG sites [1] and this variability was largely concentrated within genic regions [2]. In humans, numerous inter-individual differences of DNA methylation were also reported [3]–[6] and, importantly, the methylomes of monozygotic twins were shown to diverge during their lifetime [7]. Measuring this diversity at a genome-wide scale extended what had been observed earlier at individual loci in mice, where the level of transgene methylation was shown to strongly vary between laboratory strains [8], [9]. However, natural epigenomic variability is not restrained to DNA methylation. DNase-seq profiles of cell-lines from human families revealed ∼10,000 sites that were polymorphic in their chromatin signature [10] and it is likely that a significant fraction of them is not associated with DNA methylation differences but with other regulatory hallmarks. Natural variability was also reported at the level of high-order chromatin structure, when distinct *A. thaliana* accessions were compared for their level of genome compaction in response to light [11]. Finally, histone acetylation profiles also varies, as we previously described in a comparison of two unrelated wild strains of *S. cerevisiae* [12].

Unlike DNA variants that are irreversible and therefore tractable, epigenotypes are thought to be largely labile (i.e. able to change their state) on time scales ranging from seconds to multiple generations [13]. When the spontaneous epimutation rate of DNA methylation was estimated in 30-generations mutation accumulation lines of *A. thaliana*, it was found to be several orders of magnitude higher than the rate of DNA variation [14], [15]. Moreover, chromatin signatures not only change spontaneously but also in response to environmental conditions [16]. Various environmental factors have the potential to exert this effect. Temperature, for example, can induce dramatic epigenetic changes in plants. In the normal life cycle of many species, experiencing winter cold is essential for flowering later in spring: the *FLC* locus, whose expression prevents flowering, becomes silenced by a well-described mechanism after several weeks of vernalization (for a review, see [17]). In addition, extreme and stressful temperatures may be experienced, in which case the chromatin state of *A. thaliana* repetitive sequences can change to alleviate their silencing [18]–[20]. The response to subtle temperature variations was also shown to depend on the proper incorporation of histone variant H2A.Z [21]. In addition, specific extracellular signals such as hormones in animals can also trigger chromatin reprogramming at target loci, and the pathways involved provide many routes by which chromatin can sense environmental conditions. To a broader extent, diet represents a set of factors able to induce epigenome modifications [22]. Feeding animals with altered amounts of methyl donors can induce methylome reprogramming [23]. Such treatments have illustrated how environmental conditions may stably print epigenotypes across generations. In mice for example, reprogramming was observed in adult offsprings of *males* that had been on specific diets [24], [25].

**Figure 1:**
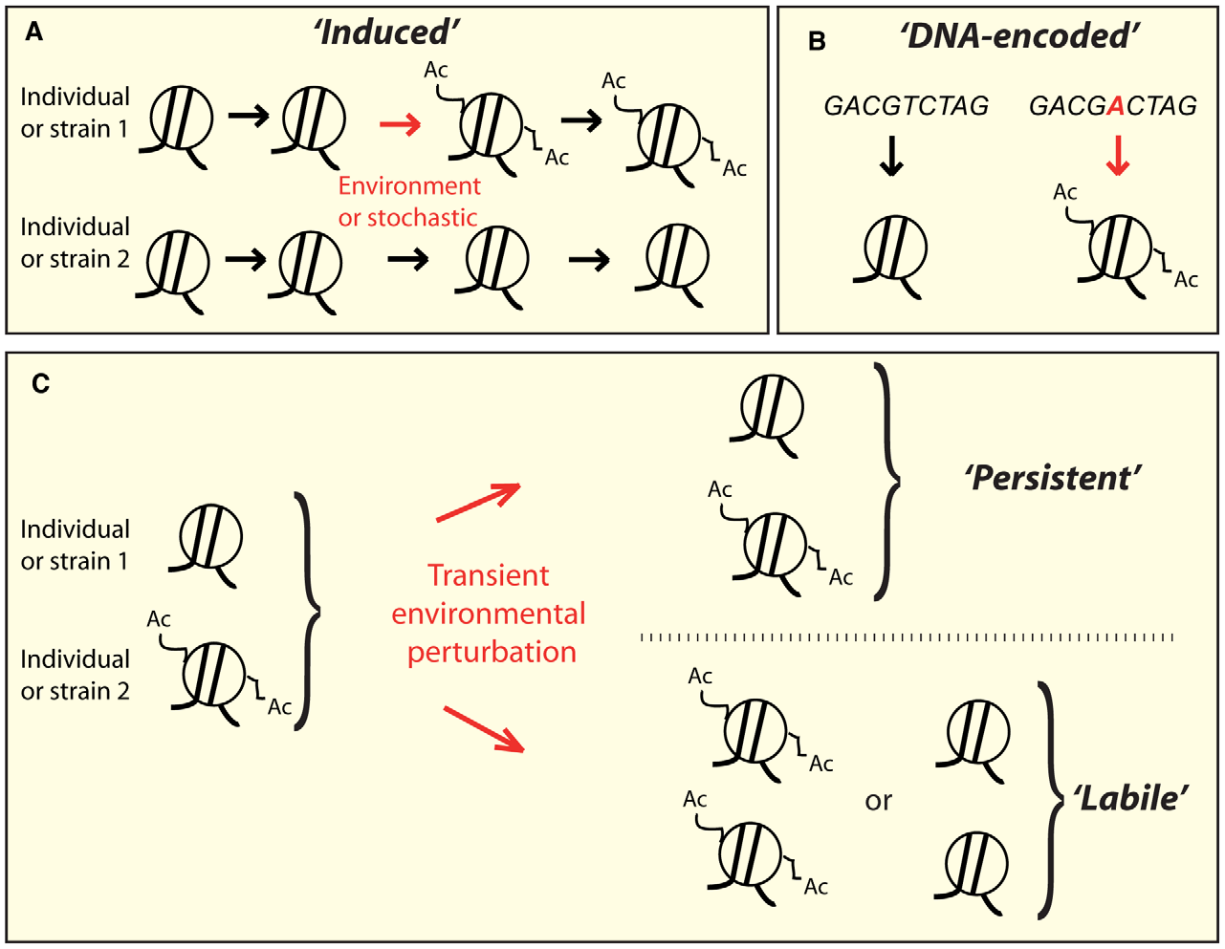
Conceptually distinct classes of epi-polymorphisms. A) *Induced* SNEPs are defined here as inter-strain differences that arose from a stochastic or environmentally-induced epigenetic change. B) *DNA-encoded* SNEPs are genetically determined by differences in the DNA sequence. C) After individuals have undergone perturbing environmental conditions, the SNEPs initially present may be lost (called *labile*), or remain (called *persistent*).

In the particular case of chromatin acetylation, direct coupling between epigenetic signatures and energy metabolism (obviously related to diet) is known to happen at least at three levels. First, sirtuins are known to deacetylate histones and a number of other proteins in a NAD^+^-dependent manner [26], [27]. Secondly, the level of Acetyl-CoA, which donates the acetyl group transferred to histones, can vary according to glucose availability and efficient metabolism [28]. And thirdly, carbonyl compounds can inactivate class I Histone Deacetylases (HDAC) by alkylation of two cysteine residues [29]. And beyond dietary effects, some environments contain natural HDAC inhibitors such as Trichostatin-A (TSA) produced by *Streptomyces platensis*, or butyrate, a natural product of the intestinal flora [30]. Thus, individuals may harbor personalized epigenomes because they have experienced a specific history of past environmental exposures or stochastic transitions (Figure 1A).

Alternatively, epi-polymorphisms can be influenced by DNA variations that modify chromatin regulations, either in *cis* (i.e. locally) or in *trans* (i.e. distantly) [31]. Well-known examples of *cis*-modifiers are transposon insertions [32], [33], whose regional effects on chromatin states have been the basis for extremely informative genetic screens in yeast (as reviewed in [32]). In humans, several heritable disorders are caused by trinucleotide repeat expansions that perturb chromatin states locally [34]. One striking example is the non-coding repeat region of the *FMR1* gene, where moderate expansions mediate hyper-acetylation of the locus and increased mRNA levels, resulting in Fragile X Tremor Ataxia Syndrome [35], whereas larger expansions induce chromatin silencing, decreased gene expression, and Fragile X Mental Retardation Syndrome [36]. The very few known *trans*-acting genetic modifiers of chromatin states are sequence changes within chromatin modifying enzymes [6], [11], but other DNA polymorphisms may also act in *trans* by affecting the activity of upstream regulators of chromatin modifying machineries. The numerous examples of DNA-encoded chromatin differences suggest that individuals may harbor distinct epigenotypes simply as a result of their different genetic content (Figure 1B).

We previously identified thousands of yeast nucleosomes carrying differential levels of H3K14 acetylation between two wild *S. cerevisiae* strains (BY and RM) [12]. Following this previous study, we define here *Single Nucleosome Epi-Polymorphisms (SNEPs)* as the intra-species variations of the level of an epigenetic mark carried on a nucleosome. The polymorphic mark may be any histone post-translational modification or the incorporation of a histone variant. A SNEP for one such mark then corresponds to the preferential presence of the mark at one nucleosomal position in some individuals or strains as compared to others. Consequently, SNEPs of various epigenetic marks may be carried on the same nucleosome. By tracking H3K14ac SNEPs, we describe here both an experimental reprogramming experiment and the genetic architecture of H3K14 acetylation variation. The results show that some epi-polymorphisms are reprogrammed after a transient perturbation of chromatin states whereas others persist, and this persistence can, at least partly, be explained by genetic determinants encoded in the DNA.

## Results

The present study focuses on one epigenetic mark, the acetylation of histone H3 at Lysine 14 (H3K14ac). For simplicity, the terms ‘SNEP’ and ‘epi-polymorphism’ are used here interchangeably to refer to H3K14ac epi-polymorphisms.

### A Transient Epi-Drug Treatment Reprograms a Subset of Epi-Polymorphisms

We previously described 5,442 SNEPs corresponding to acetylation variation between two *S. cerevisiae* strains (BY and RM). Here we assessed the stability of these epi-polymorphisms by transiently exposing the two strains to an extremely perturbing environment (Figure 1C). We sought to distinguish three types of SNEPs: *Persistent* SNEPs, corresponding to initial inter-strain differences that remained significant after the perturbation; *Labile* SNEPs, corresponding to original inter-strain differences that significantly changed after the perturbation; and *Induced* SNEPs, corresponding to inter-strain differences that appeared after the perturbation.

**Figure 2:**
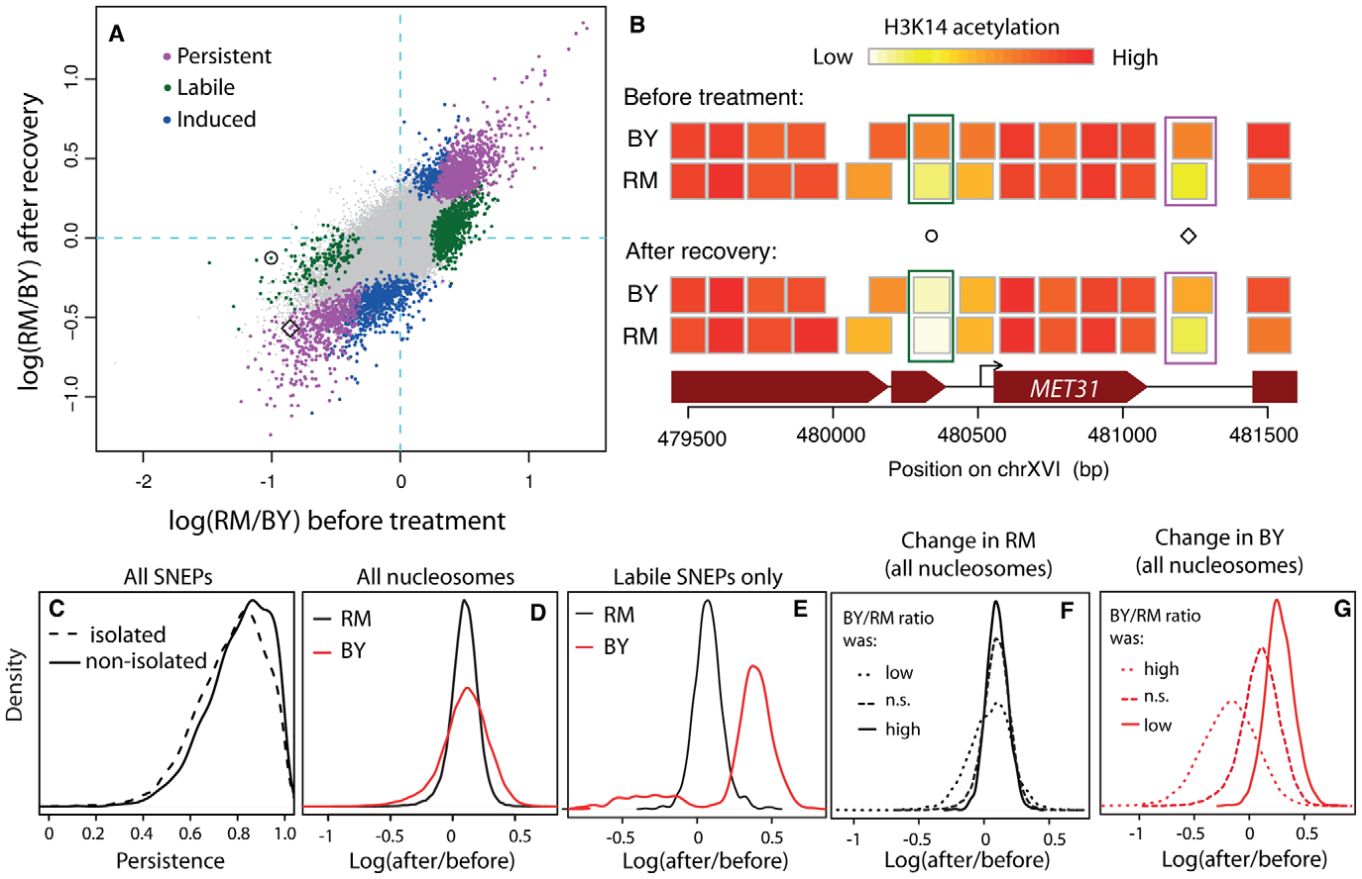
Reprogramming of SNEPs after a transient exposure to TSA. (A) Inter-strain differences in H3K14 acetylation after recovery from TSA treatment (y-axis) compared to original inter-strain difference (x-axis). Each dot represents one nucleosome. Persistent, labile and induced SNEPs are colored in magenta, green and blue, respectively. All other nucleosomes are represented by small grey dots. Circle and diamond correspond to SNEPs shown in B). (B) Examples at the *MET31* locus. Nucleosome positions (rectangles) are colored according to their level of H3K14 acetylation in BY and RM strains, before TSA treatment (top) and after recovery (bottom). Circle, a labile SNEP that was significant before TSA treatment (*P* = 3.4×10^−8^) but no longer after recovery (*P* = 0.5). Diamond, a persistent SNEP that was significant both before treatment (*P* = 1.2×10^−13^) and after recovery (*P* = 1.2×10^−7^). (C) Persistence was defined as 1−|log_2_(RM/BY)_before tratment_−log_2_(RM/BY)_post recovery_| and is shown for ‘isolated’ and ‘non-isolated’ SNEPs, which correspond to cases where no or at least one flanking nucleosome was also a SNEP, respectively (Wilcoxon test: *P*<2.2×10^−16^). (D–G) Effect of treatment and recovery in each strain. The x-axis represents the logratio of H3K14 acetylation after recovery from TSA versus before treatment, for every nucleosome considered. (D) Distributions of this logratio value measured in the RM strain (black) and the BY strain (red) in all nucleosomes. The two distributions are centered, showing a similar average effect in the two strains. The larger dispersion of the red curve indicates that more nucleosomes were reprogrammed in the BY strain. (E) Same as (D) but when only nucleosomes corresponding to labile SNEPs are considered. This shows that the reprogramming in the BY strain is not symmetric, with a majority of reprogrammed SNEPs having gained acetylation. (F) The black density curve of (D) was decomposed into three categories of nucleosomes according to the BY/RM ratio of acetylation before treatment: ‘*n.s.*’, nucleosomes that were not initially SNEPs, ‘*low*’ and *‘high’*, nucleosomes that were initially SNEPs with preferential acetylation in RM or in BY, respectively. (G) Similarly, the red density curve of (D) was decomposed into the same three categories. The fact that the distributions are shifted indicates that SNEP call prior to treatment is predictive of the treatment effect in the BY strain.

BY and RM cells were treated with high concentrations of TSA for 4–5 generations. As expected, this treatment caused a bulk increase of H3K14 acetylation in both strains (Figure S1). Cells were then washed and let grown for over 20 generations in standard medium lacking TSA. After this recovery period, the global level of H3K14 acetylation had returned to normal in both strains and we examined again inter-strain differences at single-nucleosome resolution, using chromatin immunoprecipitation and hybridization on whole genome tiling arrays, as previously described [12]. The protocol was applied on biological triplicates for each strain. Inter-strain acetylation ratios before treatment and after recovery were highly correlated (Figure 2A, Spearman *r* = 0.7).

We performed two complementary statistical analyses on the data. First, we specifically searched for induced and persistent SNEPs. To this end, we applied our previously described SNEP detection algorithm (*NucleoMiner*) to the newly generated dataset (see Methods). At a False Discovery Rate (*FDR*) of *0.0001*, we detected 2,379 SNEPs after recovery. Interestingly, 898 of them were new ones: for these nucleosomes, the level of K14 acetylation was not significantly different between the strains before treatment. They were unlikely false negatives, because detection power was higher in the initial search than after recovery from TSA (12 versus 6 microarrays used). Rather, these induced SNEPs illustrate that epi-polymorphisms may indeed result from new environmental exposures. Interestingly, 524 of the 898 induced SNEPs were ‘isolated’, i.e. their two flanking nucleosomes were not SNEPs after treatment and recovery. Of these, 436 were initially in a context where neither of the flanking nucleosome was a SNEP. This specificity illustrates that SNEPs can be induced at precise nucleosomes and not necessarily on consecutive ones.

Of the 5,442 SNEPs originally detected in normal conditions [12], 1,481 were also significant post-recovery. All of them except one had the same directionality (i.e. same strain showing increased acetylation) before treatment and after recovery and these were therefore called ‘persistent’ (Figure 2A, 2B).

The remaining 3,961 initial SNEPs could be ‘labile’, but many of them may simply be false negatives that escaped detection post recovery. We therefore applied a different test to reliably search for cases of lability: we tested for all nucleosomes if the inter-strain acetylation ratio had changed (see Methods). This was the case for 4,484 nucleosomes (*FDR = 0.001*). Among these, 1,076 belonged to the list of nucleosomes containing initial SNEPs and we therefore qualified these SNEPs as ‘labile’ (Figure 2A, 2B). These labile SNEPs did not represent cases of high experimental noise, as they were not necessarily those with low initial statistical significance (Figure S2). In conclusion, three different types of acetylation epi-polymorphisms (induced, persistent and labile) could be detected in large proportions.

We previously reported that for ∼50% of SNEPs, no acetylation variation was detectable on their flanking nucleosomes [12] (see Figure 2B for an example). Here we observed that these ‘isolated’ epi-polymorphisms globally had reduced persistence (Figure 2C, Wilcoxon *P<10^−15^*) and contained more labile SNEPs than expected (51% versus 35% among non-isolated, *P<10^−15^*, χ^2^ test). This suggests that epi-polymorphisms carried on specific single nucleosomes are less stabilized than those established on consecutive nucleosomes.

The mechanism(s) by which labile SNEPs are established and lost remain unknown. However, when confronting our data to a published map of histone turnover rates [37], we observed that labile SNEPs corresponded to nucleosomes of faster histone replacement, as compared to persistent SNEPs (*P = 0.003*, see Text S1). This suggests that the increased dynamics of molecular replacement at these positions contributes to SNEP lability. In addition, we also observed an increased persistence among nucleosomes located within protein-coding genes or located within regions of conserved DNA sequence (Figure S3).

The reprogramming experiment presented here was designed to test the stability of SNEPs and not the effect of treatment in each strain. Assessing precisely the amount of reprogramming within each strain would require a dataset where all samples prior and post treatment are processed in parallel, by the same experimenter, using common batches of reagents. This was not the case here, and confounding experimental factors would likely bias any statistical inference of reprogramming within each strain. However, we made interesting observations when inspecting the fold change of acetylation between the levels before treatment and the levels after recovery. First, the mean fold change across all nucleosomes was similar between the two strains (Figure 2D). This is consistent with the similar levels of bulk acetylation seen on whole protein extracts (Figure S1). Secondly, the fold change in the BY strain presented elevated variability between nucleosomes, as compared to the RM strain (high variance in Figure 2D). This higher variability does not correspond to higher experimental error in the BY samples, as the between-replicates variance was similar between the two strains (Table S1). This suggests that more nucleosomes were reprogrammed in the BY strain than in the RM strain. To specifically look at this possibility, we plotted the distribution of fold changes in the 1,076 labile SNEPs, where reprogramming occurred. This highlighted a strong asymmetry in BY, with a majority of labile SNEPs having gained acetylation in this strain (Figure 2E). There are at least three possible interpretations of this. First, TSA may have imposed a stronger chromatin hyperacetylation in BY than in RM. Secondly, the BY strain may have recovered badly from treatment, with a chromatin remaining at an artificially high acetylation level despite the long recovery time. Alternatively, the BY strain may initially have had many nucleosomes with low levels of acetylation, which were reset to ‘normal’ levels by exposure to TSA. In the first two cases, the observed gain of acetylation is not expected to target specific nucleosomes. In contrast, in the latter case, the nucleosomes that were reprogrammed should correspond to those initially identified as poorly acetylated in BY. In other words, the presence of a SNEP before treatment should predict the treatment effect. To see if such a prediction could be made, we considered three classes of nucleosomes on the basis of observations made before treament only: those initially SNEPs as BY hypo-acetylated, those initially SNEPs as BY hyper-acetylated, and those not initially SNEPs. We then compared the extent of fold change between these three categories of nucleosomes. The classification was not predictive of the fold change in the RM strain (Figure 2F), but it was highly predictive of the effect in the BY strain (Figure 2G). This observation suggests that the BY strain possessed many nucleosomes that were initially hypoacetylated and predisposed to resetting at a higher level.

### Genetic Dissection of H3K14ac Epigenomic Variation

**Figure 3:**
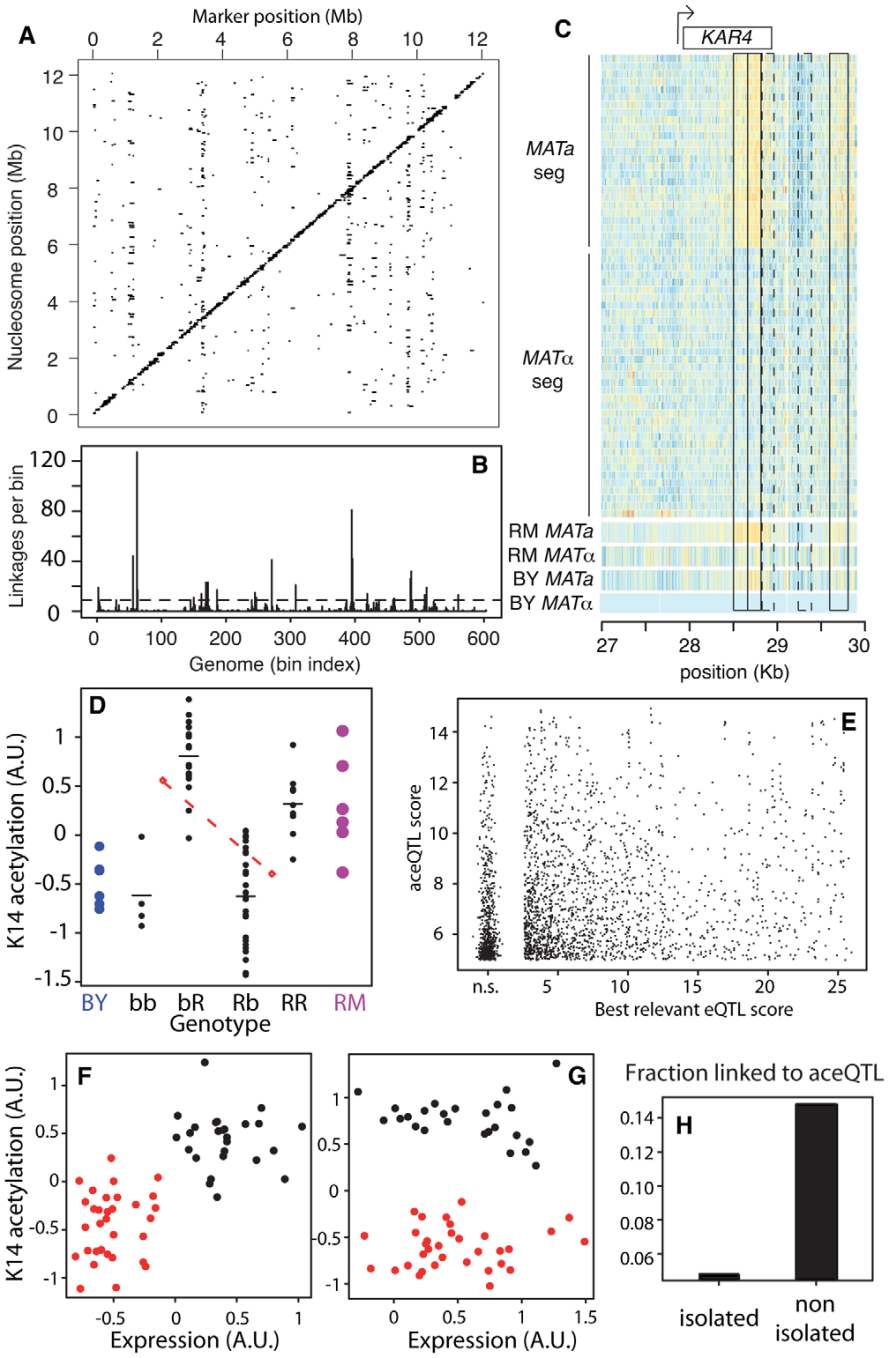
Genetic dissection of epigenomic variations. (A) Genome x Epigenome map of genetic regulations. Each dot represents a significant genetic linkage (*FDR = 0.03*) between a marker located on the x-axis and acetylation of a nucleosome located on the y-axis. Dots on the diagonal reflect *cis* regulations. (B) Distribution of *trans* regulations across aceQTL positions. Bin size: 20 Kb. Dash line: significant enrichment (*P*<0.05, see Methods). (C) Epigenomic profiles of H3K14ac at the *KAR4* locus. Color reflects ChIP-chip intensity relative to BY *MATα* from low (dark blue) to high (orange) at every informative probe (many per nucleosome). Frames indicate 5 nucleosomes in linkage with *MAT*, including 3 SNEPs (plain) and 2 nucleosomes not initially called SNEPs (dashed). The 60 segregants are separated by their mating type. (D) Acetylation of nucleosome VI-206633 (y-axis) is controlled by two *ace*QTLs of opposite effects. Each black dot represents one segregant with genotype as indicated on the x-axis (R:RM, b:BY, markers chrII-346634 and chrVI-213813, respectively). Horizontal bars: group means. Blue and magenta large dots represent replicates on BY and RM strains, respectively. Upper red diamond: mean acetylation value of segregants bb and bR. Lower red diamond: mean acetylation value of segregants Rb and RR. The dashed red line joining these diamonds indicates the effect of the first QTL on chrII (RM allele conferring low value), which counteracts the effect of the second QTL on chrVI (RM allele conferring high value). (E) Comparison of *ace*QTLs and *e*QTLs. For each significant aceQTL, all genes located within 10 Kb of the target nucleosome were considered and the one having highest eQTL score to the aceQTL marker was retained. Scores are nominal *−log_10_(P)*. n.s.: non significant scores were grouped together. (F) Correlated genetic segregation of acetylation of nucleosome chrX-458048 (y-axis) and expression of *SPC1* gene (x-axis) containing this nucleosome. Each dot represents one segregant, colored according to the genotype at the locus (red: BY, black: RM). (G) Same as F) but for nucleosome chrIII-28678 and gene *KAR4*, colored according to genotype at the *trans* aceQTL (*MAT* locus). (H) Fraction of successful *ace*QTL mapping for the two categories of SNEPs defined in Figure 2C.

We then investigated the genetic control of epi-polymorphisms. Using the maps of nucleosome positions previously generated for BY and RM [12], we associated every nucleosome with the nucleotide region that overlapped its position in both strains (see Methods). We then measured the level of acetylation of each of these regions in 60 meiotic segregants from the BYxRM cross [38]. This was done by culturing each segregant in standard laboratory conditions, and by performing single-nucleosome resolution chromatin immunoprecipitation as above. We defined one quantitative trait of acetylation per nucleosome, which reflected the abundance of the DNA region associated with the nucleosome in the immunoprecipitated material (see Methods). Using these trait values, we searched the genome for Quantitative Trait Loci of acetylation (*ace*QTL). A first scan was performed at a genome x epigenome scale. To do so, we selected 36,558 nucleosomes with H3K14ac heritability higher than 0.2, and for each of these we searched the entire genetic map for linkage. Calculations were done using a convenient platform called *eQTNMiner*, which was originally designed for the Bayesian Inference of nucleotide-resolution *e*QTLs [39]. *eQTNMiner* reports linkage evidence as a Bayes Factor (BF), which quantifies the relative support of the data in favor of the alternative hypothesis (there is a QTL) against the null hypothesis (there is no QTL). We recorded linkages at various Bayes Factor thresholds, and computed empirical significance of each threshold by a permutation test (Table S2, see Methods). At BF = 1000 (corresponding to *FDR = 0.034*), we found significant linkages for a total of 2,418 nucleosomes (Figure 3A). Of these, 77 were linked to 2 *ace*QTLs and all others to a single one.

We then applied a second scan to specifically search for *cis*-modifiers. For each nucleosome, linkage was searched across DNA polymorphisms located within 5 Kb. We chose this distance as a compromise between the small physical size of a nucleosome (147nt) and the usual large regions scanned for *cis*-*e*QTLs (10–50 Kb). At BF = 50 (corresponding to *FDR = 0.0007*), we found *cis*-linkages involving the control of 4,173 nucleosomes. There were 17% of SNEPs (908/5,442) for which an *ace*QTL could be found in at least one of the two scans. Given the rather small size of the segregating population examined, we can assume that some genetic linkages were missed. This fraction is therefore a lower-bound estimate of the ‘genetically encoded’ class of SNEPs. All further analysis was done on results obtained from the first scan only, as they reflect both *cis* and *trans* regulators, with effects strong enough to pass a stringent genome-by-epigenome significance level.

The number of nucleosomes controlled by each *trans*-acting *ace*QTLs varied greatly (Figure 3B). Seventeen loci, called ‘master-*ace*QTLs’ hereafter, were found to control more nucleosomes than expected by chance (Table 1, see Methods). One of them contained the locus controlling the cell mating type (*MAT*), which encodes different transcriptional co-factors in BY(*MAT* ***α***) and RM(*MAT* ***a***). Of the 148 nucleosomes controlled by this locus, 83 had a marked acetylation difference between BY***α*** and RM***a*** that could be detected without replicated experiments. To directly test if *MAT* accounted for the associated acetylation variation, we performed two additional ChIP-chip experiments on strains having reversed mating types (BY***a*** and RM***α***) and we tested for acetylation variation between isogenic strains differing only at *MAT*. The expected difference was observed for 69 of the 83 nucleosomes tested (*p<0.01*, see Text S1), which validated *MAT* as the responsible polymorphism underlying this master-*ace*QTL. As examples, epigenomic profiles at the *KAR4* locus are shown in Figure 3C, where the control of three SNEPs by *MAT* is apparent.

**Table 1:** Master *ace*QTLs.

*ace*QTL				Targets			Comparison to gene expression control		
Peak Position		Score(i)	candidate regulatory polymorphism(ii)	Proximal genes annotated as chromatin modifiers	# target nucleosomes	# target loci(v)	Colocalize with master-eQTL ?	Fraction of nucleosomes matching an eQTL target(iii)	Class(iv)
chrI	41 500	0.35		0	28	10	no	0.75	also *e*QTL
chrIII	76 973	1.76		0	49	15	no	0.92	also *e*QTL
chrIII	209 937	11.27	*MAT (a vs. α)*	1	148	35	yes	0.74	partial
chrV	127 339	3.79	*URA3*	1	14	9	yes	0.79	also *e*QTL
chrV	350 737	0.65		0	17	7	no	0.65	partial
chrV	553 232	3.82		2	79	60	no	0.20	*ace*QTL only
chrVI	258 538	2.86		0	17	3	no	0	*ace*QTL only
chrVIII	111 690	2.73	*GPA1-S469I*	0	24	12	yes	0.75	also *e*QTL
chrIX	30 822	2.45		0	44	14	no	0.61	partial
chrX	332 592	0.51		0	21	10	no	0	*ace*QTL only
chrXII	656 893	1.98	*HAP1 (Ty insert)*	2	124	53	yes	0.92	also *e*QTL
chrXIII	64 972	0.98		1	12	8	yes	0.08	*ace*QTL only
chrXIII	877 785	4.87		1	21	12	no	0.48	partial
chrXIV	486 860	19.86	*MKT1-D30G*	2	65	46	yes	0.75	also *e*QTL
chrXV	136 327	1.60		1	24	13	no	0.92	also *e*QTL
chrXV	193 911	5.69		1	11	9	yes	0.91	also *e*QTL
chrXVI	84 945	2.11		0	12	2	no	0.75	also *e*QTL

(i) Sum of linkage posterior probabilities across all target nucleosomes.

(ii) Based on annotations of target genes and previous *e*QTL studies [38], [47].

(iii) *Nae/Na*, where *Nae* is the number of nucleosomes targeted by this master-*ace*QTL, that are located within 10 Kb of a gene identified as an *e*QTL target of the same regulatory region; and *Na* is the total number of nucleosomes targeted by this master-*ace*QTL.

(iv) Based on the fraction of nucleosomes matching expression targets: ‘*ace*QTL only’: less than 25%, ‘also *e*QTL’: more than 75%, ‘partial’: in between. In some cases, ‘partial’ or ‘*ace*QTL only’ could be seen despite the colocalization of a master-*e*QTL because acetylation and expression control did not act on the same target loci.

(v) Target nucleosomes that were located within 1 Kb of each other were grouped into a single “locus”.

Only a small fraction (16%) of the nucleosomes controlled in *cis* were proximal to elements known to affect nearby chromatin (*Ty* transposons, *rDNA*, telomeres, *HML* and *HMR* loci). This suggests that many other causes for acetylation variability exist. Intuitively, *trans*-regulation could result from sequence variants targeting chromatin modifying enzymes. To examine this possibility, we analyzed relevant Gene Ontologies (GO). We saw that eight of the seventeen master-*ace*QTLs did not contain any gene annotated to participate in chromatin regulation (Table 1). Among all 141 *trans*-acting *ace*QTLs, 63 contained a gene with relevant annotation, which corresponded to the number expected by chance only (Text S1). Thus, *trans*-modifiers of acetylation are not necessarily restricted to chromatin modifying enzymes but may include upstream molecular players. This conclusion is analogous to the previous observation that *trans*-acting modifiers of gene expression (*e*QTLs) do not necessarily correspond to transcription factors [38].

In some cases, the genetic control of chromatin acetylation had a complex basis, such as digenic regulations by antagonistic *ace*QTLs (Figure 3D). This illustrates the quantitative nature of acetylation variation and reveals that subtle epigenomic variations can segregate as complex traits in natural populations.

### Partial Overlap between *ace*QTLs and *e*QTLs

Acetylation of H3K14 is generally a mark of active transcription [40]. However, we previously described that higher acetylation of BY/RM SNEPs did not necessarily imply an increased expression of the overlapping gene [12]. This suggests that some SNEPs do not participate in transcriptional activation while others do. Thus, one would expect that only a fraction of the genetic regulations of acetylation are concordant with the genetic regulation of gene expression. We therefore examined the overlap between *ace*QTL and *e*QTL results, taking advantage of a transcriptomic dataset previously generated on the same strains and culture conditions [38]. This was done in two steps. First, inspection of master-*ace*QTLs showed that several of them (including *MAT*) corresponded to loci previously identified as master *e*QTLs [38] (Table 1). For example, the *GPA1-S469I* polymorphism targets a G-protein α subunit and underlies expression variation of many pheromone-responsive genes [38]. This polymorphism lies at an *ace*QTL affecting 24 nucleosomes that reside within or near these target genes. *GPA1-S469I* is therefore a likely regulator of both expression and acetylation at these loci. For similar reasons, a transposon insertion altering the *HAP1* transcription factor is likely a regulator of both expression and acetylation of target genes (Table 1). However, the overall overlap between *ace*QTLs and *e*QTLs was only partial. For example, the *AMN1* locus on chromosome II was previously linked to the expression level of 18 transcripts and was not detected as a master QTL of chromatin acetylation here. Conversely, 10 master-*ace*QTLs were located at positions not previously associated with major transcriptional variation [38] (Table 1).

In a second step, we systematically compared *ace*QTL and *e*QTL linkages without restricting the analysis to master-*ace*QTLs. To do so, we reduced *ace*QTLs of the first scan to 2,530 non-redundant linkages (i.e. pairs of one nucleosome and one genetic marker). For every linkage between a genetic marker *m* and a nucleosome ν, we examined if a significant *e*QTL could be found between *m* and a gene located within 10 Kb of ν. For 31% of *ace*QTLs, no such concordance could be found. Note that statistical power was much higher to detect *e*QTLs than *ace*QTLs because many more segregants were used. It is therefore unlikely that these cases corresponded to false negatives. Overall, the strength of linkage was poorly correlated between *ace*QTLs and *e*QTLs (Figure 3E–3G, Figure S4). We then used the same criteria as above to re-examine master-*ace*QTLs and classify them based on the fraction of their linkages that matched *e*QTLs (Table 1). Of the 17 master-*ace*QTLs, nine clearly corresponded to *e*QTLs, four had partial concordance and four did not affect the expression level of genes proximal to the target nucleosomes. Notably, expression of *KAR4* was not affected by *MAT* alleles (Figure 3G). Our genetic dissection therefore unravelled the coexistence of two types of H3K14 acetylation epi-polymorphisms, one type associated with transcriptional variation and one disconnected from it. Although it is difficult to precisely estimate their relative proportions, the results argue that in at least 30% of cases, genetic polymorphisms modulate chromatin acetylation without altering gene transcription levels.

### Antagonism between SNEPs Reprogramming and Genetic Control

**Figure 4:**
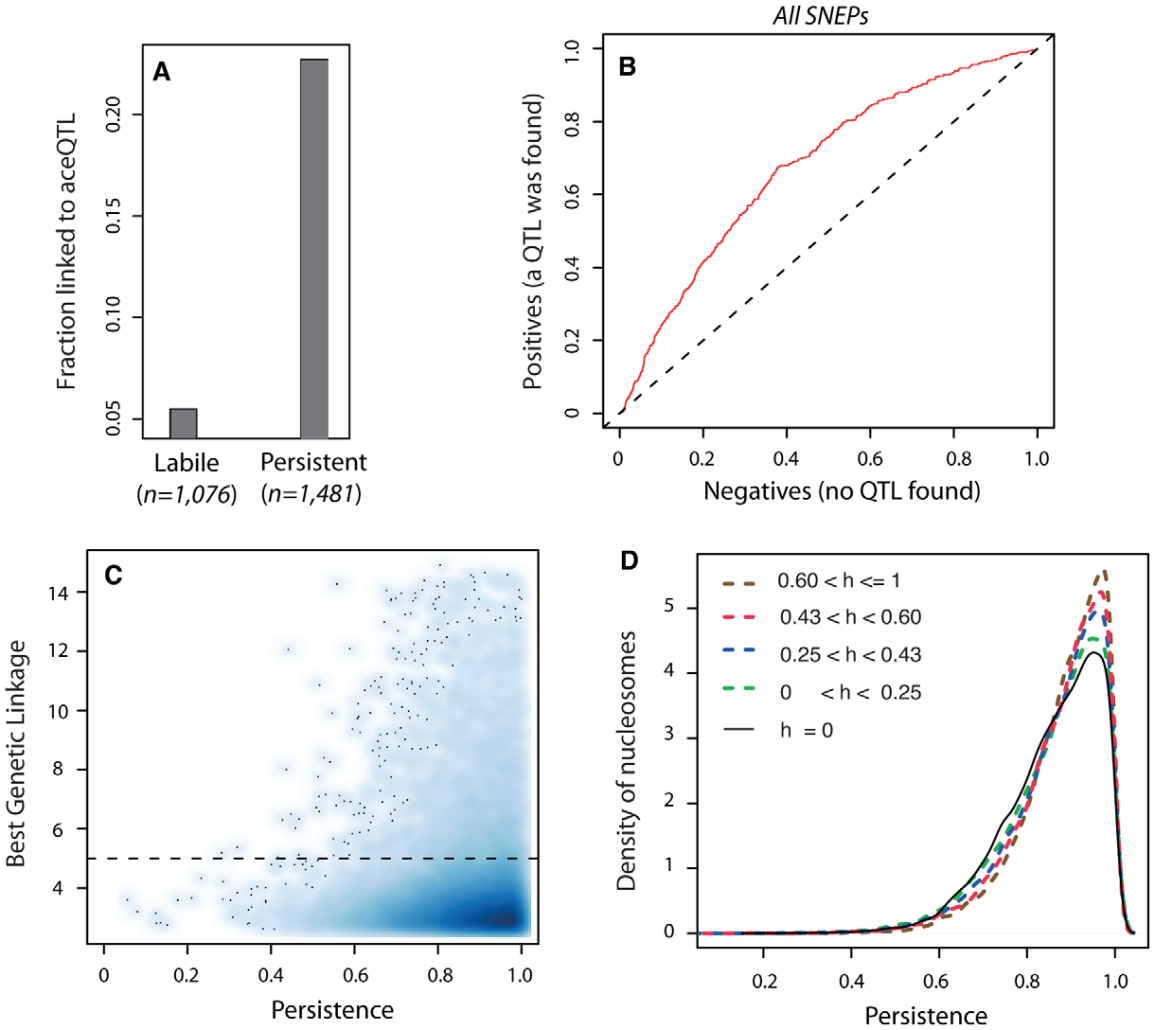
Genetic control antagonizes epigenetic lability. A) Fraction of successful *ace*QTL mapping among labile (*n* = 1,076) and persistent (*n* = 1,481) SNEPs. B) Receiver Operating Curve (ROC) analysis of frequency of *ace*QTL found among SNEPs with increasing persistence. Deviation from the diagonal shows that high persistence correlates with elevated rate of *ace*QTL discovery. C) Smooth scatter plot of all nucleosomes. X-axis: persistence value of nucleosomes, which reflects the degree of conservation of inter-strain acetylation ratio across the TSA treatment (as in Figure 2C). Y-axis: highest *−log_10_(P)* genetic linkage score found on the genome for the acetylation of this nucleosome. High linkage scores are found exclusively at high persistence. Dashed line: *ace*QTL significance threshold. To allow for identification of outsiders, dark dots represent individual data points (nucleosomes) from areas of lowest densities. D) Nucleosomes were classified according to the genetic heritability *h* of their acetylation level in the BYxRM cross. Class sizes were *n* = 14,199 nucleosomes for *h* = 0, and *n* = 11,123 nucleosomes for each category of positive *h*. Curves represent distributions of persistence for each category. A shift towards higher persistence is observed with increasing heritability values.

When epi-polymorphisms result from DNA-encoded regulatory variation, they should persist (e.g. be maintained or return to their initial state) across extreme environmental perturbations because their causative variants do. We sought to test this principle by comparing the results obtained on SNEP persistence through temporary TSA exposure with the genetic properties of *ace*QTL control. We first examined the success rate of *ace*QTL mapping when searching for regulators of ‘isolated’ or ‘non-isolated’ SNEPs. More *ace*QTLs were found for SNEPs carried on consecutive nucleosomes (Figure 3H). Note that this enrichment does not imply that *ace*QTL targets are necessarily clustered: for 60% of nucleosomes controlled by an *ace*QTL, none of the flanking nucleosome was in linkage with the same *ace*QTL locus. However, finding more *ace*QTL for clustered SNEPs was concordant with the increased persistence of these SNEPs (Figure 2C). We therefore directly examined if the presence of genetic regulators correlated with the level of environmental persistence. Accordingly, *ace*QTLs were found 4 times more often for persistent SNEPs than for labile SNEPs (Figure 4A). Consistently, a Receiver Operating Curve applied to all SNEPs showed that persistence was strongly associated with successful *ace*QTL mapping (Figure 4B). In addition, if high environmental persistence is explained by strong genetic control, then it should correlate with high genetic linkage score. We therefore represented the strength of genetic linkage as a function of environmental persistence, which confirmed the expected trend (Figure 4C).

Finally, genetic linkage results alone may sometimes not reflect the strength of genetic determinism. For example, if numerous QTLs with small individual contribution altogether control the acetylation value of a nucleosome, then none of them may be found despite a complete overall genetic determinism. Similarly, a complete control by epistatic or antagonistic genetic loci may not be detected. However, even in such complex genetic cases, the overall determinism can still be estimated by the genetic heritability of the acetylation trait in the segregating population. We therefore examined heritability itself, and found that increasing heritability values were unambiguously associated with gradual shifts towards higher persistence (Figure 4D). Thus, genetic determinism was indeed correlated with elevated environmental persistence, regardless of the complexity of the underlying control.

## Discussion

This study reveals that H3K14ac epi-polymorphisms are not equally sensitive to environmental reprogramming. Some of them can be lost after temporary perturbations while others persist. This persistence clearly correlates with the presence of genetic determinants that encode epi-polymorphisms in the DNA. This genetic control is complex and resembles architectures previously described for *e*QTLs, with both *cis* and *trans* regulators and the presence of master regulators affecting numerous targets.

Importantly, our results further highlight the quantitative nature of the variation of acetylation levels. At any given time, a nucleosome of a given cell is or is not acetylated. Thus, acetylation is sometimes considered a discrete variable. However, the average acetylation level of this nucleosome across a population of cells *is* quantitative, because it depends on the number of cells that carry the acetylation mark, which corresponds to an equilibrium state of the population resulting from many biochemical reactions. The fact that this level varies as a complex trait shows that *ace*QTLs change the proportion of cells that are acetylated at their target nucleosome. In other words, *ace*QTLs are genotypes that modify the probability that a given nucleosome is acetylated in a given cell at a given time. How this happens will probably remain unknown until new technologies are developped to interrogate single nucleosome states in single cells. Also, the quantitative variability studied here is different from several epimutations described in plants where strong silencing of large chromosomal domains is established by a combination of many molecular and structural changes.

Whether the genetic control of chromatin variability also controls the level of nearby gene expression appears to be context specific. In humans, Gibbs *et al.* did not find any consistent overlap between expression Quantitative Trait Loci (*e*QTL) modifying the transcriptome of brain tissues and Quantitative Trait Loci modifying the methylome of these tissues (*meth*QTL) [5]. In contrast, Bell *et al.* reported a clear consistency between *e*QTL and *meth*QTL in HapMap lymphoblastoid cell lines [6]. Here we observed that about 70% of *ace*QTLs linkages overlap with *e*QTLs. The remaining fraction of *ace*QTLs could correspond to regulations of non-coding transcripts, which were not interrogated by our study. Alternatively, given our previous association between SNEPs and transcriptional plasticity [12], it is possible that some *ace*QTLs modify the chromatin in a way that manifests only upon transcriptional stimulation. In other words, genetic modifiers could increase K14 acetylation of a locus, which would then become more responsive to transcriptional activation or repression upon specific conditions. In such cases, *ace*QTLs could participate in gene x environment interactions by creating epi-polymorphisms that personalize the way the genome responds to the environment.

We proved that the *MAT* locus affects chromatin acetylation of many target loci. This locus determines the cell's mating type by dictating specific transcriptional programs. The *MATα* allele encodes two regulatory proteins: α1, which activates α-specific genes, and α2, which represses expression of **a**-specific genes. The *MAT* ***a*** allele encodes the a1 protein only, which heterodimerizes with α2 in diploid ***a***/*α* cells to form a repressor of haploid-specific genes. How specific transcriptional programs are established in **a** and *α* cells has been the focus of many studies, revealing the interplay with chromatin acetylation regulation at specific target promoters. In *α* cells, the cooperative binding of α2 and Mcm1 recruits the Tup1-Ssn6 repressor, which is known to interact with several histone deacetylases [41]–[43]. In **a** cells, α-specific genes are occupied by Sum1 which is known to recruit the NAD+-dependent histone deacetylase Hst1 to repress transcription [44]. In our study, some loci controlled by *MAT* displayed an epigenomic profile totally predictable given the known transcriptional control. This was the case for the *BAR1* gene for example, which encodes a secreted protease specifically expressed in **a** cells. The chromatin signature of the entire locus was affected by the mating type. *MAT* ***a*** strains displayed occupancy and acetylation intensities typical of highly expressed genes [40], with a marked nucleosome-free region near the transcription start site, and a high and low level of H3K14 acetylation in the first and second half of the coding region, respectively (Figure S5). However, other loci controlled by *MAT* displayed unexpected patterns of chromatin variation. One such example was the *KAR4* locus, which encodes two forms of a transcription factor essential for nuclear fusion during mating. The long form is expressed in mitotically growing cells, and the short form is induced in response to pheromone from a transcriptional site about 30 nucleotides downstream the first ATG [45]. Our study revealed marked differences between *MAT* ***a*** and *MATα* growing cells in the 3′ part of the gene, which were not accompanied by differential transcriptional levels (Figure 3C and 3G). How **a** cells maintain elevated H3K14 acetylation on two nucleosomes at the end of the *KAR4* coding region remains to be identified. It is possible that **a** and α cells do not use the same strategy to maintain the locus transcriptionally active and responsive to pheromone. Comparing Ste12, Tup1, or Sum1 occupancy between **a** and α cells might reveal some differences in this region. Alternatively, DNA replication initiated downstream *KAR4*, at the ARS304 site, could have an effect if its timing differs between **a** and α cells [46]. Another particular case of mating-type specific chromatin organization was the promoter of the *SAG1* gene, which encodes the α-agglutinin specifically expressed in α cells. The repressed state of **a** cells corresponded to nucleosome occupancy downstream the TSS, and to hypoacetylation of H3K14 specifically at the -1 nucleosome (Figure S6). These three examples illustrate that the mechanism by which *MAT* alleles affect chromatin signatures at target genes is not simple: it can affect an entire locus (*BAR1*), or a specific set of nucleosomes in the promoter (*SAG1*) or the 3′ region (*KAR4*).

More generally, the fact that *ace*QTLs were not preferentially found at sites coding for chromatin modifying enzymes may seem counterintuitive: one could expect that DNA polymorphisms affect chromatin states by modifying the sequences of enzymes directly involved in chromatin regulation. However, protein complexes that regulate chromatin are themselves highly regulated, and any DNA polymorphism affecting these upstream regulators has the potential to induce chromatin modification indirectly. In fact, this is what happens with *MAT* alleles: they do not code for chromatin remodelling enzymes but they determine distinct recruitments of chromatin modifiers at specific sites. This observation is very similar to results from eQTL mapping, from which we know that genetic modifiers of gene expression do not necessarily reside in direct transcriptional regulators [38]. For example, the *AMN1, GPA1, IRA2* and *MKT1* yeast genes were all validated as *e*QTL players but they do not encode direct regulators of transcription [38], [47]. These polymorphisms affect gene expression by perturbing regulatory networks upstream of transcriptional machineries. The results presented here suggest that *ace*QTLs likely follow a similar rule: causative polymorphisms may reside not only within chromatin modifying complexes but also in their upstream regulators.

We show that a transient environmental change imposed by TSA treatment can reprogram a subset of H3K14ac epi-polymorphisms: numerous new SNEPs were induced, and numerous initial SNEPs were lost. An important consideration is that TSA imposed a perturbation but did not necessarily saturate the acetylation of H3K14 on all nucleosomes. In normal conditions, H3K14 acetylation levels result from a balance between the activity of histone acetyltransferases (HATs) and deacetylases (HDACs). In *S. cerevisiae*, at least three HATs are known to acetylate Lysine 14 of Histone H3: Gcn5p [48], [49], Sas3p [50], and Hpa2p [51], and deacetylation of Lysine 14 can be attributed to HDACs of all three classes: Hos3p and Rpd3p of class I [52], [53], Hda1p of class II [41] and Sir2p of class III [54]. TSA is known to induce a bulk hyperacetylation by inhibiting the activity of a subset of these HDACs: while Rpd3p and Hda1p are sensitive, Hos3p and Sir2p remain active. Thus, the perturbation applied in our experiment did not necessarily saturate K14 acetylation on the entire chromatin. In addition to the direct effect of TSA on HDACs that deacetylate H3K14, the treatment may have perturbed this lysine residue indirectly. The very slow growth in presence of TSA (not shown) suggests that cells profoundly reshaped molecular profiles during treatment, with possible consequences on the regulations of HATs and HDACs.

The reprogramming observed preferentially corresponded to a gain of acetylation in the BY strain, with a majority of labile SNEPs corresponding to hypo-acetylated nucleosomes in the BY strain that returned to levels comparable to those of the RM strain. An intuitive interpretation of this asymmetry would be that TSA was more efficient to induce hyperacetylation in BY than in RM. The strains are probably not equally sensitive to TSA, given the two previously mapped QTLs of growth fitness in the presence of TSA that segregate in the BYxRM cross [55]. However, the possibility that BY suffered a more pronounced hyperacetylation does not explain why only a subset of nucleosomes were preferentially reprogrammed. Alternatively, the strains may differ in their recovering efficiency. Although after 20 generations all HDAC complexes are young enough to consider they never bound the chemical inhibitor, it is still possible that the chromatin of the BY strain did not fully return to equilibrium. Then again, why would an incomplete recovery target preferentially a subset of nucleosomes? Our observation that the nucleosomes affected are largely those initially hypoacetylated suggests a third and complementary interpretation: the BY strain may have accumulated hypoacetylation ‘epimutations’ that were cured by the treatment. BY is a strain that has been maintained in laboratories for decades and is known to possess many deleterious mutations that would likely be counter-selected in the wild. Our results raise the possibility that it has also drifted at the epigenetic level, and it will be very exciting to test this hypothesis in future experiments.

More generally, it will be essential to question the origin of the ‘labile’ SNEPs: those which gained but also those which lost acetylation in BY, and the few where the change happened in RM. Theoretically, the differences in these epigenotypes may have occurred any time between the initial divergence of the strains and the last hours before the stocks were frozen in our laboratory. In other words, our study identified their lability but not their origin and age. A related question is how stable are labile and newly induced SNEPs: how harsh a treatment is needed to reprogram them? If some ‘labile’ SNEPs are old, they have been maintained for a long time and one would expect them to be stable unless extreme environmental perturbations are experienced, like in our TSA-based assay. Likewise, it is possible that additional SNEPs could have been modified if we had applied a stronger or longer treatment. As mentioned above, class III HDACs such as Sir2p are not inhibited by TSA, and other SNEPs would probably be called ‘labile’ if an inhibitor of sirtuins was used instead of TSA. In contrast, some ‘labile’ SNEPs may be very unstable and might also disappear after a prolonged but unperturbed culture. It will therefore be interesting to monitor the dynamics of SNEP appearance and loss in unperturbed conditions. A time-course experiment tracking the H3K14ac epigenome of one strain over long culture periods would help determine its stability.

How and for how long were new SNEPs induced despite the fact that the treatment applied was the same for the two strains? As mentioned above, this can possibly result from a difference in the way the strains respond to the treatment, and this difference might or not be genetically encoded. Although our experiments were not designed to address this, it is also possible that epi-polymorphisms arise stochastically in particular environments regardless of the genetic background. This has been suggested by a recent study where the methylome of isogenic mice fed with high levels of methyl precursors was tracked over generations [23]. This treatment was shown to increase inter-individual epigenome diversity, although the diet itself was common to all animals. Thus, induced epi-polymorphisms may reflect not only differences in the history of past environmental exposures, but also genetic or stochastic differences in the way individuals reprogram their epigenome in response to specific environments.

We observed a clear correlation between environmental persistence and genetic control of acetylation variation. Importantly, the two datasets (reprogramming and QTL mapping) were generated and analysed independently: at different dates, by different experimenters, the former using the parental strains only and the latter using the segregants. Thus, we believe that this correlation truly reflects the robustness of DNA-encoded epi-polymorphisms to environmental reprogramming. However, our observations do not imply that all cases of epi-polymorphism persistence result from their anchoring in DNA. It remains entirely possible that specific cases have a purely epigenetic basis. For example, H3K14 acetylation may be more robust to environmental perturbation if it is accompanied by additional epigenetic marks that are commonly associated with it, such as H3K4 di- or tri-methylation, or H3K9 acetylation [40]. If such marks drive H3K14 acetylation and are not affected by the environmental change, then persistence is ensured without a DNA-encoded control.

**Figure 5:**
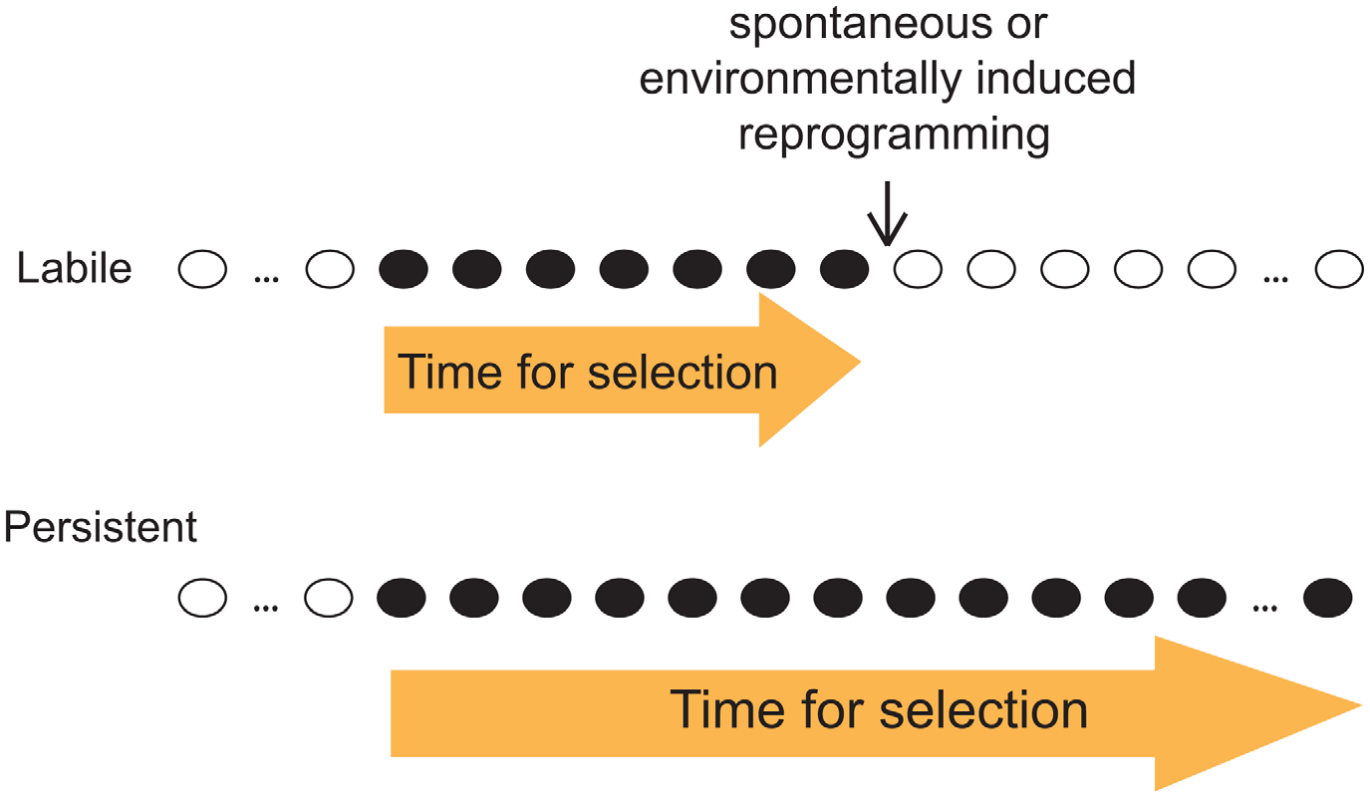
Distinct evolutionary implication of labile and persistent epi-polymorphisms. A scenario is presented where a new epigenotype (black) appears that generate a new phenotypic trait in the course of evolution. If this epigenotype is labile (up), then the trait is likely subjected to selection for a shorter time than if the epigenotype is persistent (low).

Given our observation that both labile and persistent epi-polymorphisms coexist abundantly in natural epigenomes, we emphasize the importance of the stability of epi-polymorphism in the current debate on whether and how epigenotypes contribute to evolutionary mechanisms. As outlined by B. Turner, this question is fundamental because epi-polymorphisms potentially enable environmental conditions to reprogram molecular events for a durable time. This way, “*epigenetic processes might contribute to evolutionary change, at least in part by expanding the range of phenotypic variants on which natural selection can act*” [16]. A key factor for selection to act is then the amount of time during which the ‘novel’ phenotypic variants (those generated by chromatin changes) are exposed. If too short, individuals with beneficial traits may not have time to expand in the population, especially if the phenotypic variants consist of small quantitative differences. Although our results did not link SNEPs to phenotypic traits, they suggest that the amount of time for selection to act may differ if the phenotypic variants result from labile or from persistent epi-polymorphisms (Figure 5). This duration depends on the stability of the new epigenotype and on the probability to encounter environmental conditions that change its state. If the epigenotype is robust to environmental perturbations, then the phenotype is exposed as long as other genetic or epigenetic modifiers of it are acquired. Natural selection is therefore more likely to act on phenotypic variants resulting from persistent epi-polymorphisms. Note that such high persistence can sometimes result from a full genetic control. In this case, the fact that epi-polymorphisms are involved no longer matters: selection acts on the genetic determinant regardless of the mechanism leading to the phenotype.

Importantly, loci harboring DNA-encoded epi-polymorphisms may remain highly susceptible to epigenetic regulations: as mentioned above, SNEPs represent small quantitative differences of molecular regulations, and it is likely that they do not prevent from switching between radically different epigenetic states. Thus, the prolonged duration of DNA-encoded epi-polymorphisms does not necessarily impair fitness in fluctuating environments, where adaptation requires rapid and profound chromatin remodelling at critical loci.

In addition, chromatin changes may reveal the effect of cryptic genetic variations. For example, a mutation occuring at a silenced locus can remain cryptic until silencing of the locus is alleviated. Such epigenetic alleviation might also be either labile or persistent, with different consequences on the cryptic variation: the phenotype and therefore the cryptic variation itself may be exposed to selection for a longer period of time if alleviation persists.

Altogether, our observations provide a necessary basis for the upcoming development of population epigenetics, where epi-polymorphisms of natural populations will be interpreted and possibly associated to the variation of common traits.

## Methods

### Strains and Culture Conditions

Strains used were BY4716 *MATα lys2Δ0* (called ‘BY’ in text) and BY4715 *MAT* ***a*** *lys2Δ0* derivatives of S288c, RM11-1a *MAT* ***a*** *leu2Δ0 ura3Δ0 hoΔ::KanMX* wine strain [38] (called ‘RM’ in text) and its GY689 *MATα leu2Δ0 ura3Δ0 hoΔ::KanMX amn1-A1103T* derivative (see Text S1), and 60 meiotic segregants from BYxRM previously used for eQTL mapping [38], [47]. Except for the TSA experiment, cells were grown to exponential phase in synthetic medium with 2% glucose (SDall) at 30°C as previously done for SNEP identification [12] and eQTL mapping [47].

### ChIP–chip

ChIP–chip was performed at single-nucleosome resolution as previously described, using formaldehyde fixation followed by micrococcal nuclease digestion, anti-H3K14ac antibody (Upstate #07-353) precipitation and hybridization on Affymetrix Whole Genome Yeast Tiling 4-bp resolution microarrays [12].

### Transient TSA Treatment

The following protocol was applied on 3 independent cultures for each strain. A 110 ml culture of SDall medium was inoculated at OD_600_ = 0.15 using an overnight starter culture and was grown at 30°C until OD_600_ reached 0.5–0.6. 45 ml of the culture was pelleted and frozen for later Western blot analysis (sample *t1*). TSA (Wako 204-11991) was resuspended in ethanol 50% at 45.5 mg/ml and 3.9 ml of this stock was added to the remaining of the culture (final TSA concentration: 2.6 mg/ml). The culture was kept at 30°C for 3–4 doubling times (OD_600_ = 3) and 20 ml was pelleted and frozen for Western Blot analysis (sample *t2*). Remaining cells were washed twice with 20 ml TBS1X [Tris 25 mM, NaCl 140 mM, KCl 2.5 mM, pH 7.4] and 1% of the suspension was added to 50 ml of SDall and incubated at 30°C. The next day, 10 microliters of the overnight culture was transfered to 50 ml fresh SDall medium and incubated at 30°C for 6 hours. This culture was used to inoculate 200 ml fresh SDall incubated at 30°C until OD = 0.9. This procedure corresponded to about 20 generations post-treatment. 25 ml of the final cell suspension was pelleted and frozen for Western blot analysis (sample *t3*) and the remaining of the culture was used for chromatin immunoprecipitation.

### Statistical Test for Lability

A matrix of raw microarray hybridization intensities was considered that contained the BY (6 arrays), RM (6 arrays), BYart (3 arrays) and RMart (3 arrays) ChIP-chip values for all probes having a single perfect match on both BY and RM genomes. Here ‘*Xart*’ correspond to strain *X* after recovery from TSA treatment (time t3 on Figure S1). This matrix was quantile-quantile normalized using *NucleoMiner* [12] and for every probe, 6 independent values of LR = log(BY/RM) were derived as well as 3 independent values of LRart = log(BYart/RMart). For all 58,694 previously aligned nucleosomes [12], we extracted relevant probes according to their physical position on the genome. The window of extraction was defined by the overlapping region between nucleosomal position in BY and nucleosomal position in RM [12], trimmed at both extremities by 12 nucleotides to avoid possible artefactual border effects. All probes having mid-position within this window were used to test against the null hypothesis of similar inter-strain difference before TSA treatment and after recovery (LR = LRart). This was done by an analysis of variance (ANOVA) based on the model *logratio*∼*tsa+probe*, where *tsa* reflects whether LR or LRart is considered and *probe* reflects the probe index. Nominal *P*-values relevant to factor *tsa* were used to derive *q*-values that account for multiple testing, by using the QVALUE package [56]. 4,484 nucleosomes showed *q*<0.001. Among these nucleosomes, 1,076 belonged to the list carrying original SNEPs and these SNEPs were called ‘labile’. Note that testing for acetylation reprogramming for each strain separately would be difficult from our dataset because the two sets of experiments (before treatment and after recovery) were done at different dates, by different experimenters. We therefore preferred to use inter-strain log-ratios to ensure a consistent comparison of the inter-strain difference within each dataset.

### Detection of Persistent SNEPs

We specifically searched for persistent SNEPs by running *NucleoMiner* on the BYart, RMart ChIP-chip dataset, together with the previously described BY and RM nucleosome mapping experiments [12]. 2,379 nucleosomes showed differential H3K14 acetylation levels at *FDR<0.001* (nominal *P*-value<4.05×10^−5^) after recovery from TSA. Note that fewer biological replicates were used after recovery (3 for each strain) than before treatment (6 for each strain), which explains the detection of fewer SNEPs (2,379 instead of 5,442). The intersection between the two lists of SNEPs corresponded to 1,481 nucleosomes that were called *‘persistent’* SNEPs (magenta dots on Figure 2A). For every nucleosome, persistence was defined as 1−|log_2_(RM/BY)_before tratment_−log_2_(RM/BY)_post recovery_|.

### *ace*QTL Mapping

We generated an extremely dense genetic map by inferring, at every SNP, a probabilistic genotype given the genotypes previously described at marker positions [47] (see Text S1). To avoid hybridization artefacts due to DNA polymorphisms, we considered only the microarray probes having a single perfect match on both BY and RM genomes. A dataset comprising 18 microarrays previously described (nucleosome mapping data and H3K14ac profiling on BY and RM replicates) [12] and the 60 ChIP-chip microarrays performed here on the BYxRM segregants was normalized by quantile-quantile normalization using the NMc2tab program of *NucleoMiner* with option *-n lqq*. We then computed estimates of nucleosome-level ChIP intensities. For every nucleosome, we considered signals from probes that were entirely contained in the overlap between nucleosome position in BY and nucleosome position in RM. These signals were further corrected by quantile normalization (to account for probe effects) and averaged using the *eqmr-fdb* command of *eQTNminer* [39] with option *-m qnorm*.

For every nucleosome, heritability of acetylation level was then computed as *h = (varS−varE)/varS*, where *varS* is the variance across all segregants and *varE* the environmental variance, estimated by the pooled variance of parental replicates. We noticed that one BY experiment had very high variation, therefore *varE* was estimated by the pooled variance of 5 BY and 6 RM ChIP-chip experiments.

Mapping of *ace*QTL was performed using the *eqmr-ftr* command of *eQTNMiner* [39] version 2.0 with default parameters on 59,936 nucleosomes (nucleosomes contained in translocated regions were not considered). A first scan was performed at a genome x epigenome level. To do so, we selected 36,558 nucleosomes with H3K14ac heritability higher than 0.2. This threshold was chosen arbitrarily in order to avoid multiplying tests on nucleosomes where linkage is unlikely to be discovered. For each of these, the entire genetic map was scanned for QTL using a Bayesian regression model, implemented in *eQTNMiner* [39],which follows the framework of Servin and Stephens [57]. The effect of individual *i* genotype at SNP *j* (g*_ijk_*) on the acetylation level of the *k*-th nucleosome (y*_ik_*) is assumed to follow a purely additive linear model: y*_ik_* = μ+a*_jkg_*×g*_ijk_*+ε*_ijk_*, where μ is the mean acetylation level of that nucleosome for individuals with g = 0, and where a*_jk_* is the additive effect of the minor allele at SNP *j*. The residual ε*_ijk_* is assumed to be normally distributed, with mean zero and variance 1/τ equal to the variance of acetylation levels within each genotype class. Let P0*_k_* denote the probability of the acetylation data Y*_k_* under the null hypothesis that there are no *ace*QTL controlling nucleosome *k* (i.e., a*_jk_* = 0 for all *j*). Similarly, let P1*_jk_* denote the probability of the acetylation data Y*_k_* under the hypothesis that SNP *j* is an *ace*QTL of nucleosome *k*. In this case, the effect size a*_jk_* is modelled as being drawn from mixtures of normal distributions centered on 0 (see below). The Bayes Factor reflecting genetic linkage between SNP *j* and nucleosome *k* is defined as BF*_jk_* = P1*_jk_*/P0*_k_* and measures the relative support for the hypothesis that SNP *j* is an *ace*QTL of nucleosome *k*, versus the null hypothesis. As suggested by Servin & Stephens [57], we assumed that the effect size a*_jk_* is drawn from mixtures of normal distributions centered on 0 with variance σ_a_^2^/τ. Specifically, we assumed a mixture of 6 normal with σ_a_^2^ = (0.05, 0.1, 0.2, 0.4, 0.8, 1.6), we computed a Bayes factor for each value of σ_a_^2^, and considered the mean Bayes factor as our summary statistics. We controlled the False Discovery Rate empirically by re-scanning 100 permuted datasets. On average, only 20,288 linkages were obtained at a Bayes Factor threshold of 1000 from a permuted dataset, while 592,368 linkages were obtained at this level from the actual data (Table S2). The list passing this *FDR = 0.034* threshold was used for further analysis. Note that many of the 592,368 linkages reflect redundant genetic information between adjacent DNA polymorphisms. In total, *ace*QTLs were found for 2,418 nucleosomes. To roughly see how many nucleosomes were controlled by two or more *ace*QTLs, we reduced the 592,368 linkages to account for linked markers: for each target nucleosome, the best QTL marker was recorded and all markers located within 100 Kb of it were discarded. This procedure was then repeated until no significant additional linkages remained. This way, 2341 nucleosomes were linked to a single *ace*QTL, 77 nucleosomes were linked to 2 distinct *ace*QTLs, and no nucleosome was linked to three or more loci.

A second scan was performed on all 59,936 nucleosomes to specifically detect *cis*-acting *ace*QTLs. Using the *eqmr-expca* command of *eQTNminer* [*39*], we applied a Principal Component Analysis and observed that the first 10 principal components represented significant general effects (as compared to eigenvalues obtained from permuted datasets) that could shade specific regulations. We therefore corrected for these effects by applying an elastic net regression on these 10 axes as implemented in *eqmr-fenet* of *eQTNminer*. The residuals were then used as the corrected traits. For each nucleosome, we used *eqmr-fcr* to search for linkages between the trait and any DNA polymorphism located within 5 Kb on each side. False Discovery Rate was controlled empirically by running 100 permutations (Table S3). We observed 235,942 linkages exceeding a Bayes Factor of 50 while only 160 were seen at this threshold from permuted datasets (*FDR = 0.0007*). In total, cis-*ace*QTLs were found for 4173 nucleosomes. 668 of these nucleosomes ( = 16%) were located within 20 Kb of a region known to affect nearby chromatin states (telomere, retrotransposon, rDNA, *HML* or *HMR*).

Finally, an *ace*QTL for nucleosome *i* and marker *m* found in the first scan was called trans-*ace*QTL if *m* was at least 50 Kb away from any cis-*ace*QTL found for *i* in the second scan.

### Definition of Master Trans-*ace*QTLs

We determined which of the trans-*ace*QTLs affect the acetylation level of a significantly high number of nucleosomes. To do so, we first reduced the results to the best linkage scores in *trans*. For each nucleosome for which a trans-*ace*QTL was found, the marker *M* with highest linkage score was recorded and all significant linkages to markers close to *M* were discarded. If additional significant trans-*ace*QTLs remained for this nucleosome, the procedure was repeated. We then segmented the genome in 20 Kb bins and counted the number of reduced trans-*ace*QTLs in each bin (Figure 3B). We tested for enrichment by considering deviation from Poisson distribution, as done before for eQTLs [38], [47]. 25 bins were significantly enriched (at least 9 target nucleosomes, *P<0.05* after Bonferroni correction), which could be concatenated to 17 non-consecutive bins. We then searched each bin for the best candidate polymorphism regulating the set of target nucleosomes: for every nucleosome *i* having a trans-*ace*QTL in the bin, we computed at every SNP *k* the posterior probability P*_i_*(*k*) that the SNP is causal. This probability can be directly computed from the output of *eQTNminer*. Let *B_i,m_* be the Bayes Factor for linkage between nucleosome *i* and SNP *m*, and *S_i_* the sum of *B_i,m_* across all *m* of the genome, then the probability is simply P*_i_*(*k*) = B*_i,k_*/S*_i_*. These probabilities were then sumed across all nucleosomes in linkage to the bin, and the best candidate was identified as the SNP maximizing this sum (indicated as ‘Score’ in Table 1).

### *ace*QTL Versus *e*QTL

To compare *ace*QTLs with *e*QTLs in a consistent way, we generated a set of *e*QTL results using the same method and same genetic map as for *ace*QTLs. Gene expression data was extracted for 4,464 genes from the “glucose condition” of Smith and Kruglyak [47]. *eQTNminer* was used to scan the genome x transcriptome space, without the hierarchical models previously described [39] and using data from 109 segregants previously generated under the same glucose medium as here [47]. This produced 2,159,456 linkages with Bayes Factor exceeding 50. One hundred permutations were run to control the FDR, which was 3.5% at this threshold. In total, *e*QTLs were found for 3,572 genes and the results were consistent with previous studies.

To then determine if *ace*QTLs could be considered as *e*QTLs, we considered all significant *ace*QTLs of the first scan. To remove redundant linkages supported by adjacent markers, we reduced the results to the best scores as described above for reducing *trans*-*ace*QTLs, leaving 2,530 *ace*QTL linkages out of the 592,368 original ones. For each one linking acetylation of a nucleosome *i* to a genetic marker *m*, we then recorded the best eQTL score between *m* and any gene located within 10 Kb of *i*. In several cases, no *e*QTL was found at a very relaxed threshold (Bayes Factor of 1) and this search was then labelled as ‘non-significant’ (Figure 3E). We considered that an *ace*QTL was not an *e*QTL if the best score found was not more significant than P<0.00125 (nominal value). This corresponds to the usual 0.01 threshold divided by 8 which is the average number of genes examined within 20 Kb of the yeast genome (4,464 * 20/12,000). Following this criterion, 790 of the 2,530 *ace*QTL linkages (31%) did not correspond to eQTL. Note that the detection power was much higher for *e*QTL than for *ace*QTL, as more segregants were used. Thus, it is unlikely that we missed relevant *e*QTLs at this relaxed threshold.

We also addressed the reciprocal question of whether *e*QTLs were *ace*QTLs. Redundant *e*QTLs supported by adjacent markers were removed as above. For each *e*QTL found at *FDR = 0.035* between a marker *m* and a gene *g*, we considered all *ace*QTL scores between *m* and any nucleosome located with 10 Kb of *g* and recorded the best one. When no *ace*QTL was found at the relaxed threshold of BF = 1, then this search was called “non significant” (Figure S4).

To estimate whether master trans-*ace*QTLs correspond to master *e*QTLs (Table 1), we proceeded as follows. For each master trans-*ace*QTL controlling the acetylation levels of a set of nucleosomes ν_i_, let *m* be the best candidate polymorphism as defined above. For each nucleosome ν_i_ we examined all genes located within 10 Kb and asked whether at least one of them was a significant *e*QTL target of *m*. The fraction of nucleosomes ν_i_ for which this was the case was called “*Fraction of nucleosomes matching an eQTL target*”. Because many target nucleosomes ν_i_ were located close to each other, we also examined them as distinct target loci: Target nucleosomes ν_i_ that were located within 1 Kb of each other were grouped into a “locus”. For each locus, we counted the fraction of target nucleosomes for which a relevant *e*QTL linkage was found (as above). This number was then averaged across all target loci to define the “*Average fraction per locus*” indicated in Table 1. Finally, master trans-*ace*QTLs were classified as being ‘also *e*QTL’, ‘*ace*QTL only’ or ‘partial’ based on whether this fraction was higher than 75%, lower than 25%, or in between, respectively.

### ROC Analysis

Figure 4B was obtained by sorting the 5,442 original SNEPs by their persistence across the transient TSA treatment (defined above). A Receiver Operating Curve (ROC) was then built: ‘positive’ SNEPs were the ones for which at least one significant *ace*QTL was found, as this corresponds to the expectation of a genetic control underlying persistence; ‘negative’ SNEPs were those for which no *ace*QTL was found. Fraction of positives and negatives were computed at increasing persistence values.

### Data Accession

All ChIP-chip raw data is available from ArrayExpress (http://www.ebi.ac.uk/arrayexpress/) under accession numbers E-MTAB-575 and E-MTAB-1025. Additional processed data files are available from our web site: http://www.ens-lyon.fr/LBMC/gisv/snep/

## Supporting Information

Figure S1 Western-Blot of whole protein extracts from BY and RM strains. Times *t1* and *t2* correspond to prior and immediately after 8-hours of treatment with 0.03 mg/ml Trichostatin-A, respectively. A sample of treated cells was then used to inoculate normal medium and let grown for ∼20 generations for recovery (time *t3*).
(PDF)

Figure S2 SNEP lability is not associated with poor significance. A) Dot plot of all nucleosomes representing their score (Y-axis) for having a different inter-strain ratio of K14ac before and after treament (ANOVA test described in methods) as a function of their score (X-axis) for being initially a SNEP (ANOVA test described in Nagarajan et al. 2010). These scores are −log_10_(*P*) where *P* is the statistical significance. *R*: Spearman correlation coefficient. Red-circled dots: nucleosomes corresponding to labile SNEPs, i.e. being a SNEP because they have on the X-axis a *P*-value lower than the 9.27×10^−6^ cutoff defined in Nagarajan et al. 2010, and being labile because they have on the Y-axis a score corresponding to a *q*-value lower than the 0.001 cutoff used to call lability (see Methods). B) Receiver Operating Curve (ROC) of the ‘labile’ vs. ‘non-labile’ calls as a function of initial SNEP significance. An association between ‘labile’ calls and poor initial significance would produce a curve significantly above the diagonal, and not below as observed.
(PDF)

Figure S3 Persistence of different classes of nucleosomes. Each panel represents the distributions of persistence values (as in Figure 2C) of all nucleosomes splitted into two classes. A) Within *versus* outside a region coding for an mRNA transcript. Higher persistence is seen for nucleosomes within coding regions (Wilcoxon Mann-Whitney P<2.2×10^−16^) B) Within *versus* outside a region of conserved DNA sequence (as extracted from UCSC website http://genome.ucsc.edu/, using table *phastConsElements* for track *MostConserved*). Higher persistence is seen for nucleosomes within conserved regions (Wilcoxon Mann-Whitney P<2.2×10^−16^).
(PDF)

Figure S4 Comparison of *e*QTLs to *ace*QTLs (reverse analysis as Figure 3E). For each significant *e*QTL, all nucleosomes located within 10 Kb of the target gene were considered and the one having highest *ace*QTL score to the *e*QTL marker was retained. Scores are nominal *−log_10_(P)*. n.s.: very low, non significant scores were grouped together.
(PDF)

Figure S5 Epigenomic profiles of nucleosome occupancy and H3K14ac at the *BAR1* locus. Color on the upper lane reflects MNase-chip intensity logratio between RM and BY, indicating differences of nucleosome occupancy. Color on all other lanes reflects H3K14ac ChIP-chip intensity relative to BY *MATalpha*. In all cases, low and high values correspond to dark blue and orange, respectively, at every informative probe (many per nucleosome). The 60 segregants are separated by their mating type. Region labelled ‘1’ is depleted of nucleosome in RM-*MATa* as compared to BY-*MATalpha* (upper lane), which explains the low signal of H3K14ac ChIP in MATa strains in this region. Regions ‘2’ and ‘3’ have a more precise positioning of nucleosomes in RM than in BY (periodicity of orange bands in upper lane). *MATa* strains show a pronounced H3K14 acetylation in region 2 and a remarkably low H3K14 acetylation in region 3.
(PDF)

Figure S6 Epigenomic profiles of nucleosome occupancy and H3K14ac at the *SAG1* locus. Color on the upper lane reflects MNase-chip intensity logratio between RM and BY, indicating a difference of nucleosome occupancy in the region labelled ‘1’. Color on all other lanes reflects H3K14ac ChIP-chip intensity relative to BY *MATalpha*. In all cases, low and high values correspond to dark blue and orange, respectively, at every informative probe (many per nucleosome). The 60 segregants are separated by their mating type. Arrow: transcription start site. A nucleosome is labelled ‘2’ and corresponds to a SNEP in genetic linkage to *MAT*. We see that the mating type affects both nucleosome occupancy in region ‘1’ and H3K14 acetylation of nucleosome ‘2’.
(PDF)

Table S1 Standard deviations of probe-level intensities among the BY and RM triplicates post recovery.
(DOC)

Table S2 Numbers of genetic linkages found in the genome x epigenome scan at various False Discovery Rates (FDR).
(DOC)

Table S3 Numbers of cis-*ace*QTLs found in dedicated scan at various FDRs.
(DOC)

Table S4 GO terms used to extract genes related to chromatin modifying activity.
(DOC)

Text S1 Additional detailed methods.
(DOC)
