# Supplementary figures and images for: Genetic Modifiers of Chromatin Acetylation Antagonize the Reprogramming of Epi-Polymorphisms
Source: PLoS Genet. 2012 Sep 20;8(9):e1002958. doi: 10.1371/journal.pgen.1002958 (PMC3447955; doi:10.1371/journal.pgen.1002958)

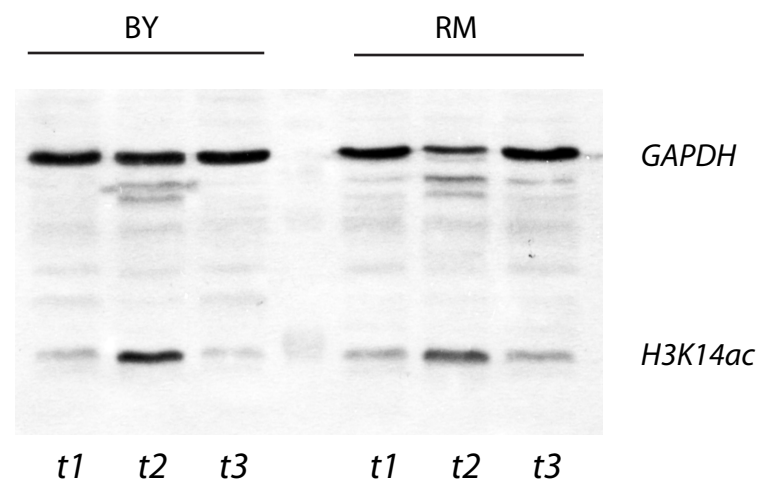

Figure S1

Supplement: Figure S1 — Western-Blot of whole protein extracts from BY and RM strains. Times t1 and t2 correspond to prior and immediately after 8-hours of treatment with 0.03 mg/ml Trichostatin-A, respectively. A sample of treated cells was then used to inoculate normal medium and let grown for ∼20 generations for recovery (time t3). (PDF) [file pgen.1002958.s001.pdf]

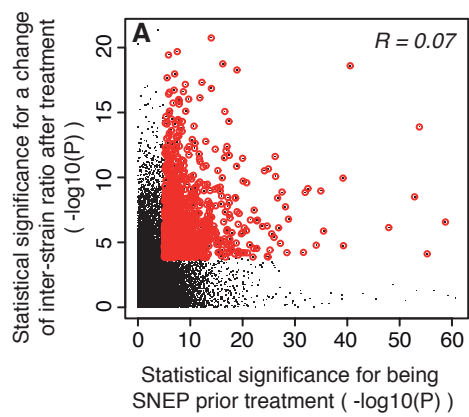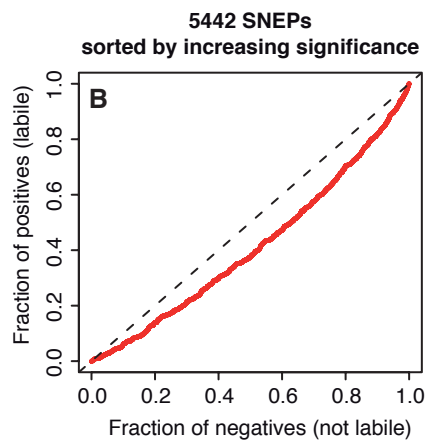

Figure S2

Supplement: Figure S2 — SNEP lability is not associated with poor significance. A) Dot plot of all nucleosomes representing their score (Y-axis) for having a different inter-strain ratio of K14ac before and after treament (ANOVA test described in methods) as a function of their score (X-axis) for being initially a SNEP (ANOVA test described in Nagarajan et al. 2010). These scores are −log10(P) where P is the statistical significance. R: Spearman correlation coefficient. Red-circled dots: nucleosomes corresponding to labile SNEPs, i.e. being a SNEP because they have on the X-axis a P-value lower than the 9.27×10^−6^ cutoff defined in Nagarajan et al. 2010, and being labile because they have on the Y-axis a score corresponding to a q-value lower than the 0.001 cutoff used to call lability (see Methods). B) Receiver Operating Curve (ROC) of the ‘labile’ vs. ‘non-labile’ calls as a function of initial SNEP significance. An association between ‘labile’ calls and poor initial significance would produce a curve significantly above the diagonal, and not below as observed. (PDF) [file pgen.1002958.s002.pdf]

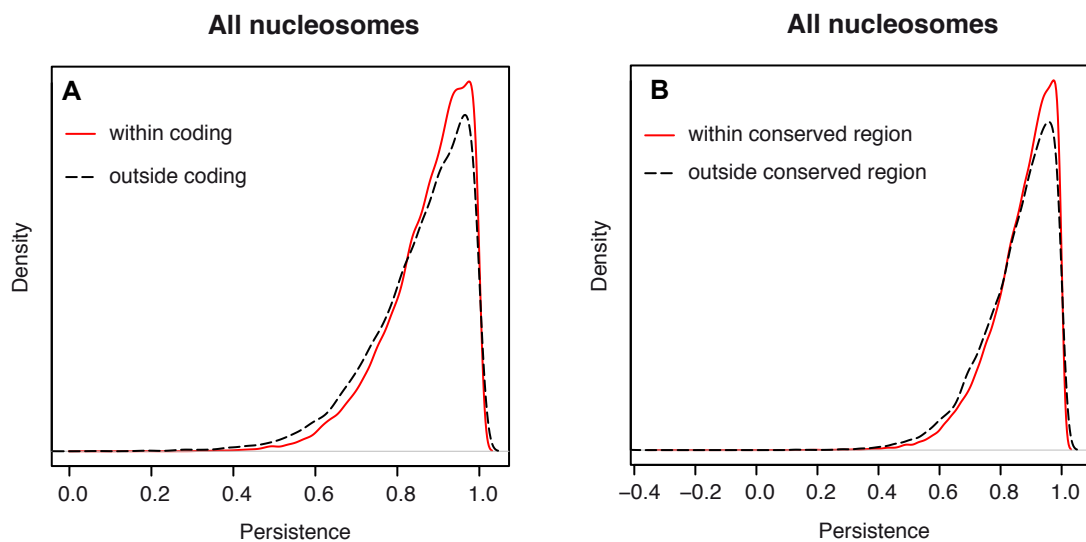

Figure S3

Supplement: Figure S3 — Persistence of different classes of nucleosomes. Each panel represents the distributions of persistence values (as in Figure 2C) of all nucleosomes splitted into two classes. A) Within versus outside a region coding for an mRNA transcript. Higher persistence is seen for nucleosomes within coding regions (Wilcoxon Mann-Whitney P<2.2×10^−16^) B) Within versus outside a region of conserved DNA sequence (as extracted from UCSC website http://genome.ucsc.edu/, using table phastConsElements for track MostConserved). Higher persistence is seen for nucleosomes within conserved regions (Wilcoxon Mann-Whitney P<2.2×10^−16^). (PDF) [file pgen.1002958.s003.pdf]

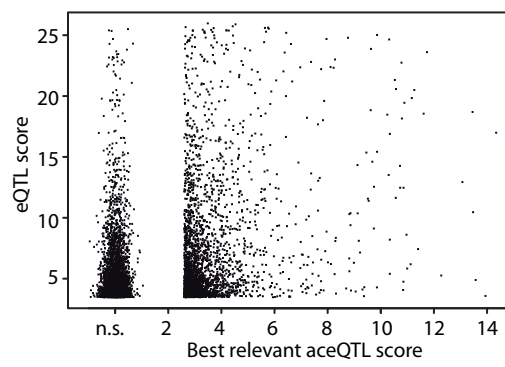

Figure S4

Supplement: Figure S4 — Comparison of eQTLs to aceQTLs (reverse analysis as Figure 3E). For each significant eQTL, all nucleosomes located within 10 Kb of the target gene were considered and the one having highest aceQTL score to the eQTL marker was retained. Scores are nominal −log10(P). n.s.: very low, non significant scores were grouped together. (PDF) [file pgen.1002958.s004.pdf]

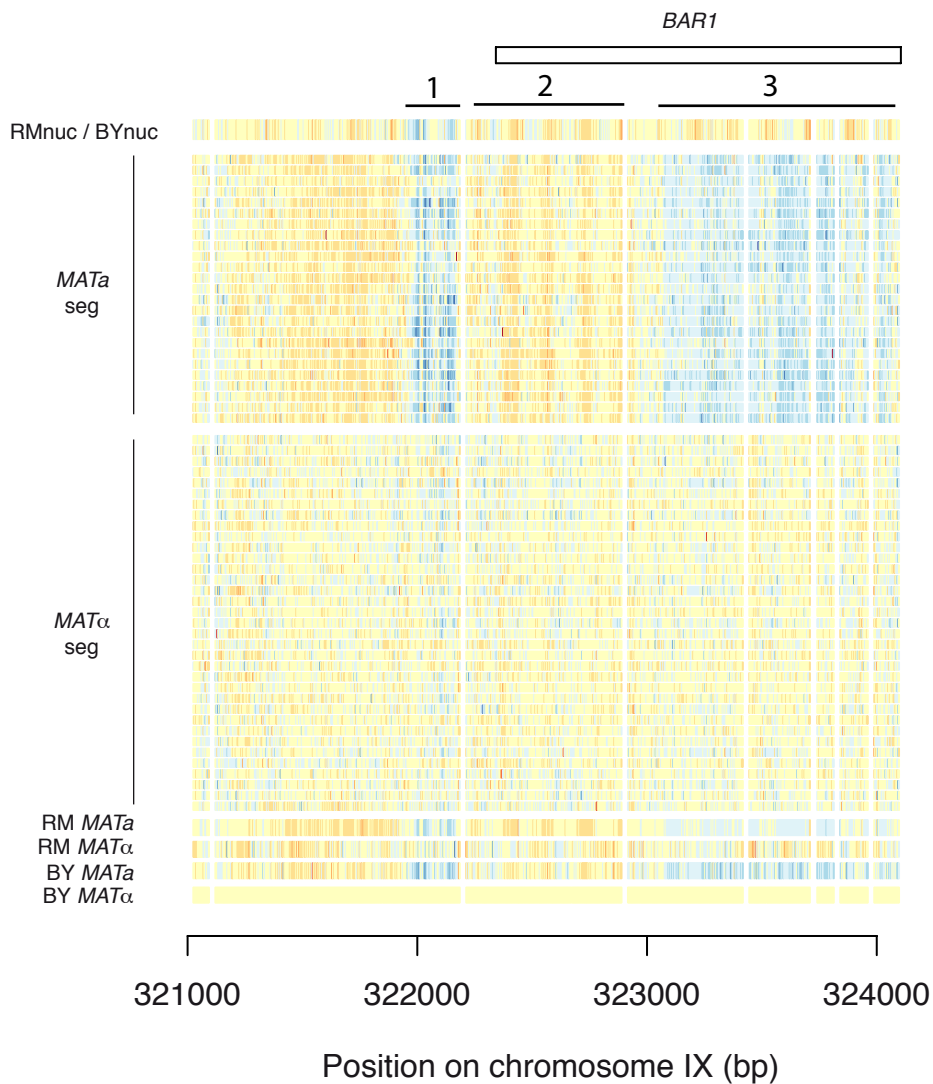

Figure S5

Supplement: Figure S5 — Epigenomic profiles of nucleosome occupancy and H3K14ac at the BAR1 locus. Color on the upper lane reflects MNase-chip intensity logratio between RM and BY, indicating differences of nucleosome occupancy. Color on all other lanes reflects H3K14ac ChIP-chip intensity relative to BY MATalpha. In all cases, low and high values correspond to dark blue and orange, respectively, at every informative probe (many per nucleosome). The 60 segregants are separated by their mating type. Region labelled ‘1’ is depleted of nucleosome in RM-MATa as compared to BY-MATalpha (upper lane), which explains the low signal of H3K14ac ChIP in MATa strains in this region. Regions ‘2’ and ‘3’ have a more precise positioning of nucleosomes in RM than in BY (periodicity of orange bands in upper lane). MATa strains show a pronounced H3K14 acetylation in region 2 and a remarkably low H3K14 acetylation in region 3. (PDF) [file pgen.1002958.s005.pdf]

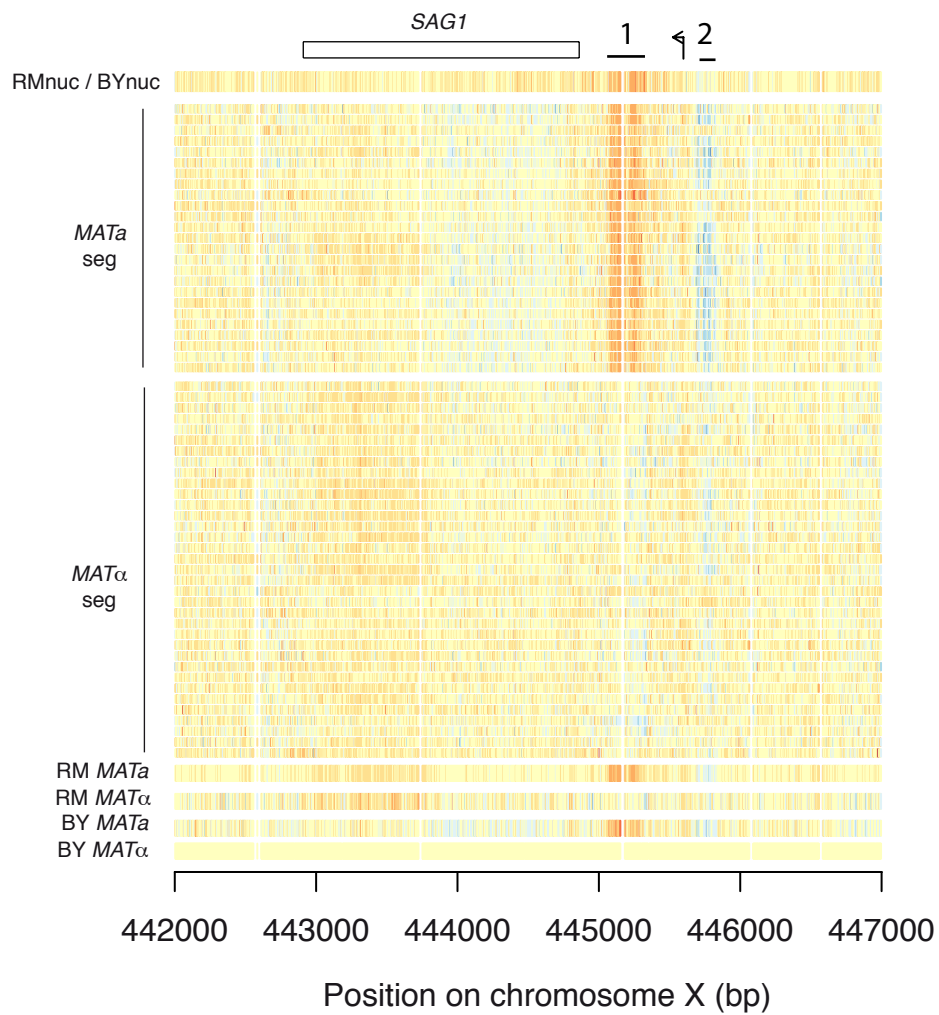

Figure S6

Supplement: Figure S6 — Epigenomic profiles of nucleosome occupancy and H3K14ac at the SAG1 locus. Color on the upper lane reflects MNase-chip intensity logratio between RM and BY, indicating a difference of nucleosome occupancy in the region labelled ‘1’. Color on all other lanes reflects H3K14ac ChIP-chip intensity relative to BY MATalpha. In all cases, low and high values correspond to dark blue and orange, respectively, at every informative probe (many per nucleosome). The 60 segregants are separated by their mating type. Arrow: transcription start site. A nucleosome is labelled ‘2’ and corresponds to a SNEP in genetic linkage to MAT. We see that the mating type affects both nucleosome occupancy in region ‘1’ and H3K14 acetylation of nucleosome ‘2’. (PDF) [file pgen.1002958.s006.pdf]
