# Supplementary material for: Genetic Modifiers of Chromatin Acetylation Antagonize the Reprogramming of Epi-Polymorphisms
Source: PLoS Genet. 2012 Sep 20;8(9):e1002958. doi: 10.1371/journal.pgen.1002958 (PMC3447955; doi:10.1371/journal.pgen.1002958)
Supplement: Table S1 — Standard deviations of probe-level intensities among the BY and RM triplicates post recovery. (DOC) [file pgen.1002958.s007.doc]

**Table S1.** Standard deviations of probe-level intensities among the BY and RM triplicates post recovery.

|  | Min | 1^st^ quartile | Median | Mean | 3^rd^ quartile | Max |
| --- | --- | --- | --- | --- | --- | --- |
| sdBY | 0.00 | 0.15 | 0.26 | 0.32 | 0.43 | 5.55 |
| sdRM | 0.00 | 0.17 | 0.28 | 0.34 | 0.46 | 5.61 |

Numbers are summaries of the sdBY and sdRM vectors containing 2,356,675 values (one per microarray probe). Each value is the strandard deviation of the three independent intensities (biological triplicates) observed on one strain.
