# Supplementary material for: Genetic Modifiers of Chromatin Acetylation Antagonize the Reprogramming of Epi-Polymorphisms
Source: PLoS Genet. 2012 Sep 20;8(9):e1002958. doi: 10.1371/journal.pgen.1002958 (PMC3447955; doi:10.1371/journal.pgen.1002958)
Supplement: Table S2 — Numbers of genetic linkages found in the genome x epigenome scan at various False Discovery Rates (FDR). (DOC) [file pgen.1002958.s008.doc]

**Table S2.** Numbers of genetic linkages found in the genome x epigenome scan at various False Discovery Rates (FDR).

| Bayes Factor | Observed | Expected | FDR |
| --- | --- | --- | --- |
| 1 | 8032683 | 4572810 | 0.5693 |
| 10 | 8032660 | 4572777 | 0.5693 |
| 50 | 2068206 | 612611 | 0.2962 |
| 100 | 1394965 | 270904 | 0.1942 |
| 500 | 728501 | 43766 | 0.0601 |
| 1000 | 592368 | 20288 | 0.0342 |
| 10000 | 339094 | 1652 | 0.0049 |
| 50000 | 237690 | 292 | 0.0012 |
| 100000 | 205744 | 141 | 0.0007 |
