# Supplementary material for: Genetic Modifiers of Chromatin Acetylation Antagonize the Reprogramming of Epi-Polymorphisms
Source: PLoS Genet. 2012 Sep 20;8(9):e1002958. doi: 10.1371/journal.pgen.1002958 (PMC3447955; doi:10.1371/journal.pgen.1002958)
Supplement: Table S3 — Numbers of cis-aceQTLs found in dedicated scan at various FDRs. (DOC) [file pgen.1002958.s009.doc]

**Table S3.** Numbers of cis-*ace*QTLs found in dedicated scan at various FDRs.

| Bayes Factor | Observed | Expected | FDR |
| --- | --- | --- | --- |
| 1 | 1021443 | 61021 | 5.97E-02 |
| 10 | 351051 | 1192 | 3.40E-03 |
| 50 | 235942 | 160 | 6.78E-04 |
| 100 | 205630 | 65 | 3.16E-04 |
| 500 | 153823 | 8 | 5.20E-05 |
| 1000 | 136773 | 4 | 2.92E-05 |
| 10000 | 93498 | 0.5 | 5.35E-06 |
| 50000 | 73560 | 0.05 | 6.80E-07 |
| 100000 | 65272 | 0.01 | 1.53E-07 |
