# Supplementary material for: Genetic Modifiers of Chromatin Acetylation Antagonize the Reprogramming of Epi-Polymorphisms
Source: PLoS Genet. 2012 Sep 20;8(9):e1002958. doi: 10.1371/journal.pgen.1002958 (PMC3447955; doi:10.1371/journal.pgen.1002958)
Supplement: Table S4 — GO terms used to extract genes related to chromatin modifying activity. (DOC) [file pgen.1002958.s010.doc]

**Table S4.** GO terms used to extract genes related to chromatin modifying activity.

| **ID** | **Description** |
| --- | --- |
| GO:0006333 | chromatin assembly or disassembly |
| GO:0043044 | ATP-dependent chromatin remodeling |
| GO:0016568 | chromatin modi_cation |
| GO:0006325 | chromatin organization |
| GO:0006338 | chromatin remodeling |
| GO:0031055 | chromatin remodeling at centromere |
| GO:0006342 | chromatin silencing |
| GO:0000183 | chromatin silencing at rDNA |
| GO:0030466 | chromatin silencing at silent mating-type cassette |
| GO:0006348 | chromatin silencing at telomere |
| GO:0006343 | establishment of chromatin silencing |
| GO:0006345 | loss of chromatin silencing |
| GO:0001308 | loss of chromatin silencing involved in replicative cell aging |
| GO:0031936 | negative regulation of chromatin silencing |
| GO:0061188 | negative regulation of chromatin silencing at rDNA |
| GO:0061186 | negative regulation of chromatin silencing at silent mating-type cassette |
| GO:0031939 | negative regulation of chromatin silencing at telomere |
| GO:0016569 | covalent chromatin modi_cation |
| GO:0071169 | establishment of protein localization to chromatin |
| GO:0071168 | protein localization to chromatin |
| GO:0031938 | regulation of chromatin silencing at telomere |
| GO:0034401 | regulation of transcription by chromatin organization |
