# Supplementary material for: Genetic Modifiers of Chromatin Acetylation Antagonize the Reprogramming of Epi-Polymorphisms
Source: PLoS Genet. 2012 Sep 20;8(9):e1002958. doi: 10.1371/journal.pgen.1002958 (PMC3447955; doi:10.1371/journal.pgen.1002958)
Supplement: Text S1 — Additional detailed methods. (DOC) [file pgen.1002958.s011.doc]

TEXT S1

ADDITIONAL DETAILED METHODS

**Strains.** To obtain a *MATalpha* derivative of RM, we switched the mating type of strain YEF1946 (Nogami et al. ) by transient expression of a plasmid-encoded HO endonuclease. A spontaneous diploid was made to sporulate after losing the plasmid. Tetrads were dissected to select one spore (GY689: *MATalpha leu2Δ0 ura3Δ0 hoΔ::KanMX amn1-A1103T*).

**Western Blot.** Whole-cell protein extracts were prepared by washing cells once and resuspending them in 200 microliters of extraction buffer (EB) [75mM Tris-HCl pH 6.8, 0.6% SDS, 15% Glycerol]. A similar volume of acid-washed microbeads (Sigma #G8772) was added and the mixture was vortexed for 1 hour at 4°C. Samples were then centrifuged at 13,000 rpm for 5 min at 4°C. The supernatant was transferred to a fresh tube and sonicated three time for 10 seconds on ice using a VibraCell 75185 (130W, amplitude 40%). Debris were discarded by centrifuging at 13,000 rpm for 1min at 4°C and transferring the supernatant to a fresh tube. About 50μg of proteins was migrated on a 15% SDS-PAGE gel and transferred to a 0.45μm nitrocellulose membrane. Proteins were detected using anti-H3K14ac polyclonal antibody (Upstate #07-353, dilution1:5,000), ECL anti-rabbit secondary antibody (GE Healthcare #NA9340V, dilution 1:10,000), and anti-GAPDH-HRP Goat antibody (Abcam #ab85760, dilution 1:2,000).

**Histone turnover rates of Labile and Persistent SNEPs.** We used the nucleosome turnover rates estimated by Dion et al . This study was based on two-colors Agilent microarrays (one probe every 265 bp). For each SNEP, we looked for Agilent probes contained in the BY nucleosome and extracted the corresponding turnover rate [‘G1_LAMBDA’ field of Table S2 of Dion et al]. We then applied a two-sided Wilcoxon-Mann-Whitney test against the null hypothesis of no difference between turnover rates of *Labile* and *Persistent* SNEPs (*P* = 0.003). To see if this test could be biased by the fact that clustered SNEPs tended to have higher persistence than isolated SNEPs, we applied this test again on clustered SNEPs only (distinguishing 952 persistent from 515 labile ones). The difference remained significant (*P* = 0.007), ruling out the possible bias. When considering isolated SNEPs only, the trend was still observed but no longer significant, probably because fewer observations were available (529 persistent and 561 labile ones).

**Genetic map.** A matrix of genotypes of 124 BYxRM segregants at 2820 marker positions was obtained from a previous study . These markers correspond to single feature polymorphisms (SFP) affecting hybridization intensities on Affymetrix S98 microarrays. We used this dataset to generate a genetic map with probabilistic genotypes at every DNA polymorphism known from the alignments of BY and RM genomic sequences. First, we assigned each SFP to a known sequence polymorphisms and excluded the very rare cases (3 markers) where this assignment was not possible. We then imputed recombination fractions and genotypes at every DNA polymorphism by fitting, for each chromosome, a Hidden Markov Model as previously described . The resulting map contained both observed and imputed genotypes at 49,593 polymorphic sites.

**GO analysis.** To examine if trans-*ace*QTLs could generally be explained by polymorphisms residing in known chromatin modifying enzymes, we considered all GO annotations relevant to chromatin regulation (22 terms, Table S4). These terms were associated with a total of 254 genes, called ‘chromatin relevant genes’ hereafter. In a first step, we simply counted the number of ‘chromatin relevant genes’ falling within the bins defined as master-*ace*QTLs (Table 1, column 5). In a second step, we also considered *ace*QTL linkages involving markers and nucleosomes that were at least 100Kb away from each other, or located on distinct chromosomes. There were 949 such *trans* linkages, involving 544 genetic markers. We then performed two complementary tests. First, we examined if trans-*ace*QTLs contained more ‘chromatin relevant genes’ than expected by chance. There were 80 such genes falling within 10 Kb of at least one of the 544 markers, which was exactly the average number obtained when picking 254 yeast genes at random (i.e. regardless of annotation). Conversely, we tested if the neighborhood of ‘chromatin relevant genes’ contained more trans-*ace*QTL markers than expected by chance. To do this, we first reduced the markers to 141 distinct regions. We observed that 63 of these regions were located within 10Kb of a ‘chromatin relevant gene’, which was comparable to the number obtained after re-assigning the position of the ‘chromatin relevant genes’ to random positions on the genome.

**Validation of *MAT* as master trans-*ace*QTL.** The BY and RM strains described in the main text have mating types *MATalpha* and *MATa*, respectively. We performed one H3K14ac ChIP-chip experiment on strain BY4715 (called BY *MATa* hereafter) and one on strain GY689 (called RM *MATalpha* hereafter), allowing direct validation of *MAT* as a controller of acetylation based on 6 values for BY *MATalpha*, 6 values for RM *MATa*, 1 value for BY *MATa* and 1 for RM *MATalpha*. In addition, nucleosome positioning data was available from 3 hybridizations for BY and 3 hybridizations for RM . As above, we discarded all probes having either multiple perfect matches or any mismatch on any of the two genomes. We log-transformed the raw intensity values and normalized them by quantile-quantile normalization using *NMcel2tab* program of *Nucleominer* with option *-n lqq*. The dataset was then reduced to 3 probe-level values: *Y_BYRM_ = <C_BY_> - <N_BY_>* -( *<C_RM_> - <N_RM_>*); *Y_BYaBY_ = C_BYa_ - <C_BY_>*  and *Y_RMalphaRM_ = C_RMalpha_ - <C_RM_>*; where *C_X_* was the probe intensity in one ChIP experiment of strain X and *N_X_* was the probe intensity in one nucleosome mapping experiment of strain X. For all 148 nucleosomes linking to the master-*ace*QTL containing *MAT*, data was extracted for all probes matching the nucleosome position. By considering every probe-level difference as an independent estimate of the sample’s difference, we applied three Student’s *t-*tests against the following null hypotheses.

H_0_1: “In the BY and RM samples, *Y_BYRM_* is equal to zero”

H_0_2: “The BY and BYa samples verify Y_BYaBY_ ≤ 0 in the case where <Y_BYRM_> is negative and Y_BYaBY_ ≥ 0 in the case where <Y_BYRM_> is positive”.

H_0_3: “The RM and RMalpha samples verify Y_RMalpha_ ≤ 0 in the case where <Y_BYRM_> is positive and Y_RMalpha_ ≥ 0 in the case where <Y_BYRM_> is negative”.

This way, nucleosomes where H_0_1 can reliably be rejected are SNEPs for which averaging experimental replicates still allowed the detection of BY/RM differences in the samples. Rejections of H_0_2 and H_0_3 represent successful validations of the effect of *MAT* by the BYa and RMalpha experiments, respectively. Of the 148 nucleosomes tested, H_0_1 was rejected at *p<0.01* for 83 nucleosomes. Of these, 59 and 37 were significant for rejecting H_0_2 and H_0_3, respectively and 69 were significant for rejecting either H_0_2 or H_0_3 or both at *p<0.01.*

**Annotations of nucleosomes controlled by *ace*QTLs that were not eQTLs**. The linkages annotated *n.s.* on Figure 3E corresponded to *ace*QTLs that were not eQTLs. They controlled a total of 735 nucleosomes. We interrogated this set to see if it was enriched for (or depleted from) nucleosomes that

- were located within coding regions
- were located wihin regions of conserved DNA sequence
- were +1 nucleosomes of transcribed genes

The significance of the corresponding Fisher exact test was *P*=0.06, *P*=0.7 and *P*=0.32, respectively, revealing no clear association to these annotations.

REFERENCES

1. Nogami S, Ohya Y, Yvert G (2007) Genetic complexity and quantitative trait loci mapping of yeast morphological traits. PLoS Genet 3: e31.

2. Dion MF, Kaplan T, Kim M, Buratowski S, Friedman N, et al. (2007) Dynamics of replication-independent histone turnover in budding yeast. Science 315: 1405-1408.

3. Smith EN, Kruglyak L (2008) Gene-Environment Interaction in Yeast Gene Expression. PLoS Biol 6: e83.

4. Lincoln SE, Lander ES (1992) Systematic detection of errors in genetic linkage data. Genomics 14: 604-610.

5. Nagarajan M, Veyrieras JB, de Dieuleveult M, Bottin H, Fehrmann S, et al. (2010) Natural single-nucleosome epi-polymorphisms in yeast. PLoS Genet 6: e1000913.
